# A reconstituted system reveals how activating and inhibitory interactions control DDK dependent assembly of the eukaryotic replicative helicase

**DOI:** 10.1093/nar/gkv881

**Published:** 2015-09-03

**Authors:** M. Carmen Herrera, Silvia Tognetti, Alberto Riera, Juergen Zech, Pippa Clarke, Alejandra Fernández-Cid, Christian Speck

**Affiliations:** DNA Replication Group, Institute of Clinical Sciences, Imperial College London, London W12 0NN, UK

## Abstract

During G1-phase of the cell-cycle the replicative MCM2–7 helicase becomes loaded onto DNA into pre-replicative complexes (pre-RCs), resulting in MCM2–7 double-hexamers on DNA. In S-phase, Dbf4-dependent kinase (DDK) and cyclin-dependent-kinase (CDK) direct with the help of a large number of helicase-activation factors the assembly of a Cdc45–MCM2–7–GINS (CMG) complex. However, in the absence of S-phase kinases complex assembly is inhibited, which is unexpected, as the MCM2–7 double-hexamer represents a very large interaction surface. Currently it is unclear what mechanisms restricts complex assembly and how DDK can overcome this inhibition to promote CMG-assembly. We developed an advanced reconstituted-system to study helicase activation in-solution and discovered that individual factors like Sld3 and Sld2 can bind directly to the pre-RC, while Cdc45 cannot. When Sld3 and Sld2 were incubated together with the pre-RC, we observed that competitive interactions restrict complex assembly. DDK stabilizes the Sld3/Sld2–pre-RC complex, but the complex is only short-lived, indicating an anti-cooperative mechanism. Yet, a Sld3/Cdc45–pre-RC can form in the presence of DDK and the addition of Sld2 enhances complex stability. Our results indicate that helicase activation is regulated by competitive and cooperative interactions, which restrict illegitimate complex formation and direct limiting helicase-activation factors into pre-initiation complexes.

## INTRODUCTION

Accurate DNA replication is essential for the faithful duplication of the genome (1). In all eukaryotes initiation of DNA replication is a two-step process: first, during G1 phase, MCM2–7 helicase loading occurs. During this process ORC/Cdc6 and Cdt1 recruit two MCM2–7 hexamers to replication origins. In a multi-step reaction the two MCM2–7 hexamers become loaded into a head-to-head double-hexamer around double stranded DNA (2–4). However, this MCM2–7 double-hexamer does not function as a helicase until it is activated in S-phase (1,5). The second step of DNA replication is triggered by the Dbf4 dependent kinase Cdc7 (DDK) and S-phase specific cyclin-dependent kinase (S-CDK) (6,7). These two kinases promote the assembly of a stable Cdc45–MCM2–7–GINS (CMG) complex, which represents the active replicative DNA helicase (8–10). During this reaction, which is also termed pre-initiation complex (pre-IC) formation, DNA unwinding occurs and single-stranded DNA is generated, which represents a landing pad for DNA polymerases (11).

The early steps in pre-IC formation have been analyzed *in vivo* in *Saccharomyces cerevisiae*, where it was shown that Sld3 supports Cdc45 recruitment, although its detection required crosslinking, suggesting weak interactions (12–14). Sld7, which is not essential in yeast, interacts with Sld3 and may affect Cdc45 recruitment as well (15). S-CDK functions to phosphorylate Sld2 and Sld3, and these two proteins are the essential S-CDK targets (16,17). CDK phosphorylation of Sld2 and Sld3 stabilizes Sld2-Dpb11-Sld3 interactions and promotes recruitment of GINS and polymerase epsilon to the replication origin (18).

Recent work using reconstituted systems with yeast extracts or purified proteins identified that DDK and CDK control pre-IC formation. Interestingly, it was observed that in the absence of DDK pre-IC formation is completely blocked (6,7). This is surprising, as the MCM2–7 double-hexamer represents a very large interaction surface. Importantly, DDK does not alter the overall structure of the MCM2–7 double-hexamer (19,20), instead it was suggested that DDK dependent phosphorylation of MCM2–7 generates binding sites for replication factors (5,19). To understand how DDK regulates pre-IC formation, it will be crucial to separate the complete process in individual steps, which can be interrogated in a precise manner e.g. the identification of specific protein interactions, the temporal order of complex assembly and the stability of the complexes.

Here, we studied the recruitment of helicase activation factors to replication origins using a reconstituted system that employs purified proteins from *S. cerevisiae*. We found that a kinase directed network of interactions drives complex assembly and equally complex disassembly, revealing a novel layer of regulation during pre-IC formation. Specifically, we found that the Mcm2 subunit represents a binding surface for Sld3, Cdc45 and Sld2. However, in the context of the pre-RC, only Sld2 and Sld3 can interact with the MCM2–7 double-hexamer, while Cdc45 cannot. Interestingly, competitive Sld2, Sld3 interactions with the pre-RC limit complex assembly. DDK can stabilize the Sld2/Sld3–pre-RC complex, but the complex is only short lived, indicative of an anti-cooperative mechanism. However, addition of Cdc45 to Sld2/Sld3–pre-RC greatly stabilizes the super-complex, highlighting the cooperativity of this reaction. Additionally, using a DDK bypass mutant we observed that a structural change in MCM2–7 is sufficient to form a Sld2/Sld3/Sld7/Cdc45–pre-RC complex. We suggest that anti-cooperative interactions serve as a quality control mechanism to redistribute limiting factors to preferred sites of complex assembly. On the other hand, cooperative interactions are important for complex assembly during pre-IC formation.

## MATERIALS AND METHODS

### *In vitro* Cdc45 loading assay

Pre-RCs were assembled using a one-step reaction. Fifty microliter reactions containing 40 nM ORC, 40 nM Cdc6, 40 nM Cdt1 and 40 nM MCM2–7 were pre-incubated for 10 min on ice and 10 min at 24°C in buffer I [50 mM Hepes–KOH (pH 7.5), 100 mM KGlu, 10 mM MgAc, 50 μM ZnAc, 3 mM ATP, 5 mM DTT, 0.1% Triton X-100, and 5% glycerol]. 6 nM pUC19-ARS1 plasmid and 1.5 U of human topoisomerase I (Topogen) were added to the reaction and incubated for 15 min at 24°C 950 rpm. The minus pre-RC control does contain the DNA, but not the pre-RC proteins (ORC, Cdc6, Cdt1 and MCM2–7). Afterwards the following components were added to a final concentration of: 5 nM DDK, 40 nM Sld3, 40 nM Sld2, 40 nM Sld7, 40 nM Dpb11 and 40 nM Cdc45, 5 mM ATP, 250 ng/μl BSA, 1.4 μM competitor DNA (40 bp). Due to the addition of these factors the volume of the reaction increased by 15 μl. The reactions were incubated for 1–30 min at 27°C 950 rpm. Then, KAc was added to a final concentration of 200 mM followed by a 3 min incubation at 24°C and mixing at 950 rpm. Samples were loaded on top of a spin column filled with 0.5 ml of resin (Sephacryl S400 HR, GE). After loading of the samples the columns were spun for 5 min at 1000 g. Prior to the gel-filtration step the spin columns were filled with equilibrated resin (buffer I containing 200 mM KAc, but no Triton X-100 and glycerol) and pre-spun for 5 min at 850 g. The eluate was concentrated by speed-vac prior to SDS-PAGE analysis. The experiments have been repeated at least three times.

Supplementary methods can be found with this article online.

## RESULTS

### An assay to study pre-IC assembly and function

**Figure 1.**
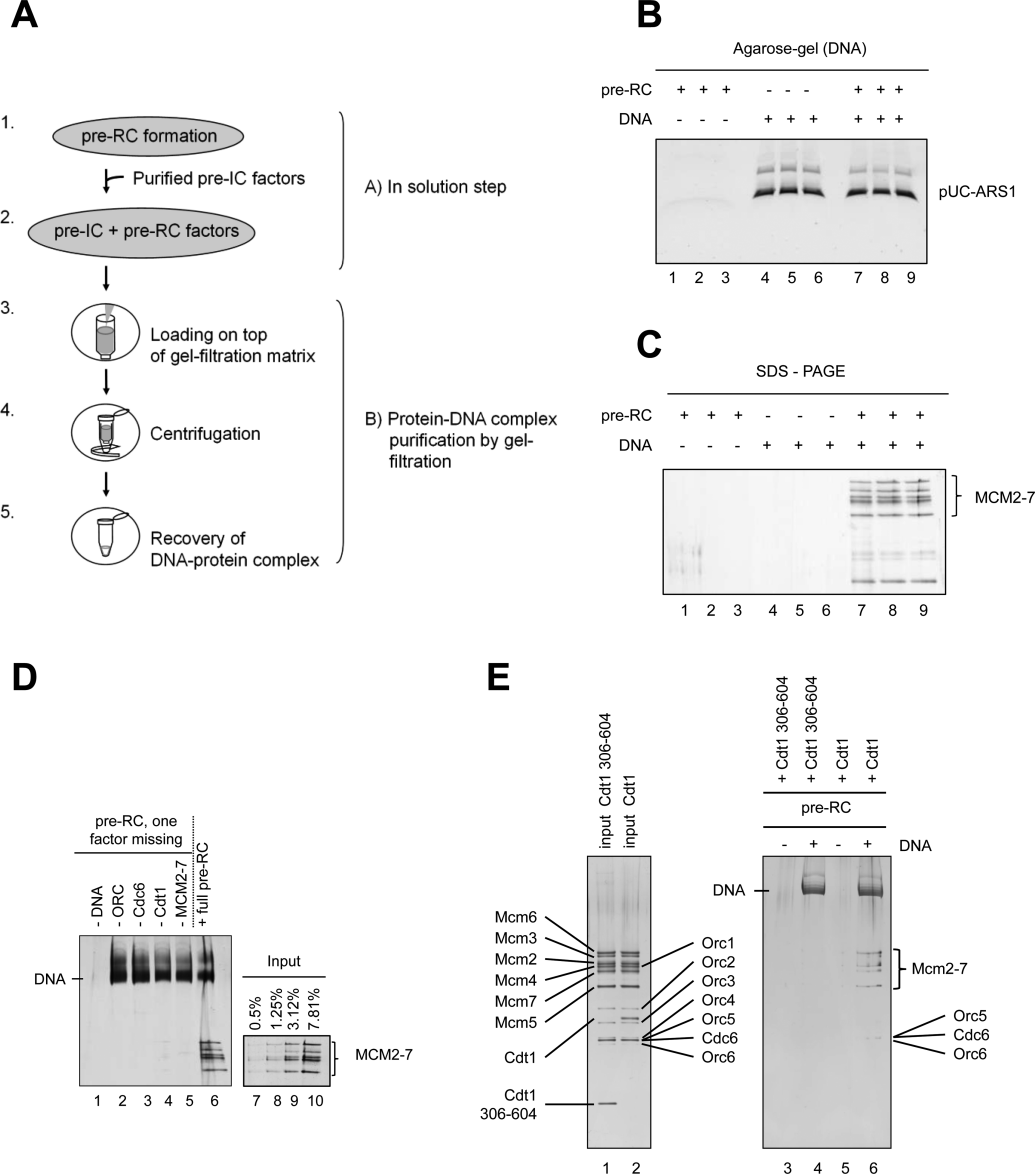
A gel-filtration based MCM2–7 loading and pre-IC assembly assay. (**A**) Illustration of the gel-filtration based method. [1] Pre-RC formation is initiated in solution. [2] Factors involved in Cdc45 loading and competitor DNA are added to the pre-RC reaction, the mixture is incubated and then high salt is added. [3] After a short incubation with high salt the mixture is added to a gel-filtration column. [4] The sample is centrifuged. [5] DNA bound protein complexes elute, while unbound proteins are retained within the gel-filtration matrix. (**B**) DNA reproducibly elutes from the gel-filtration column (lanes 4–6 and 7–9) (**C**) while pre-RC proteins are only recovered with DNA, but not in the absence of DNA (compare lanes 7–9 with 1–3), similar as described with a magnetic bead based pre-RC assay. (**D**) Pre-RC formation is highly specific. Removal of each individual factor out of the reaction blocks MCM2–7 recruitment to the pre-RC. (**E**) A Cdt1 (306–604) mutant, that facilitates MCM2–7 association, but not loading, failed to support salt stable MCM2–7 association in the absence and presence of DNA (compare lane 3 and 4 with 5 and 6). A 20% load of the proteins is shown in lanes 1 and 2. The DNA eluted from the gel-filtration column is indicated in the silver-stained gel.

The establishment of an *in vitro* reconstituted system using purified proteins is a powerful approach to study DNA replication (7,21,22). Here, we adopted a method that permits us to study the binding of individual S-phase specific replication factors to the pre-RC in the absence of magnetic beads. We used in solution assembly of protein-DNA complexes followed by a gel filtration step to purify complexes (Figure 1A). During the spin-column mediated gel-filtration the protein–DNA complexes eluted readily, while soluble proteins got trapped by the gel-filtration matrix and therefore did not elute. Previously, we have used this technique in the context of the pre-RC and electron microscopy, but the general approach is well established (2,23–26). Since MCM2–7 is a large complex (605 KDa), we employed a gel-filtration matrix that can trap protein complexes of up to 8000 KDa, but allows the elution of DNA molecules of 271 bp or larger. To eliminate non-specific protein–DNA interactions we added a competitor DNA into the reaction, which is composed of small double-stranded oligonucleotides (40 bp) that become trapped by the gel-filtration matrix. Moreover, just prior to the gel-filtration, we added high salt, which destabilizes pre-RC intermediates and non-specific protein–DNA interactions. One advantage of this system is that it does not involve a solid matrix during protein-DNA complex assembly, as a matrix can cause serious nonspecific protein interactions (2,3). Initially, we characterized the system for pre-RC assembly. We assessed the recovery of DNA and proteins in the absence of DNA, in the absence of pre-RC proteins and in the presence of DNA and pre-RC proteins (Figure 1B and C). These experiments were performed in triplicates to study the reproducibility of the assay. To understand the specificity of the assay we performed reactions lacking individual pre-RC factors. Only in the presence of all pre-RC factors MCM2–7 association with DNA was observed (Figure 1D). To test if the employed high salt wash is capable of removing associated MCM2–7, we assembled complexes with a Cdt1 (306–604) mutant, which does not support MCM2–7 double-hexamer formation (27). The Cdt1 mutant failed to support salt stable MCM2–7 interaction with DNA, showing that the high-salt indeed destabilizes associated, but not loaded MCM2–7 complexes (Figure 1E). Using the gel filtration based pre-RC assay we observed that about 3–7% of the input MCM2–7 was transformed into a high salt resistant MCM2–7 complex, which equates to 10–20% of the DNA molecules being in complex with an MCM2–7 double-hexamer (Figure 1D), similar as previously observed (2). In summary, the gel-filtration based pre-RC assay supports specific and reproducible DNA recovery and complex assembly, while the addition of high salt removes pre-RC intermediates, but not the loaded MCM2–7 double-hexamer.

### Sld2 and Sld3 interact with origin DNA in a pre-RC dependent manner

**Figure 2.**
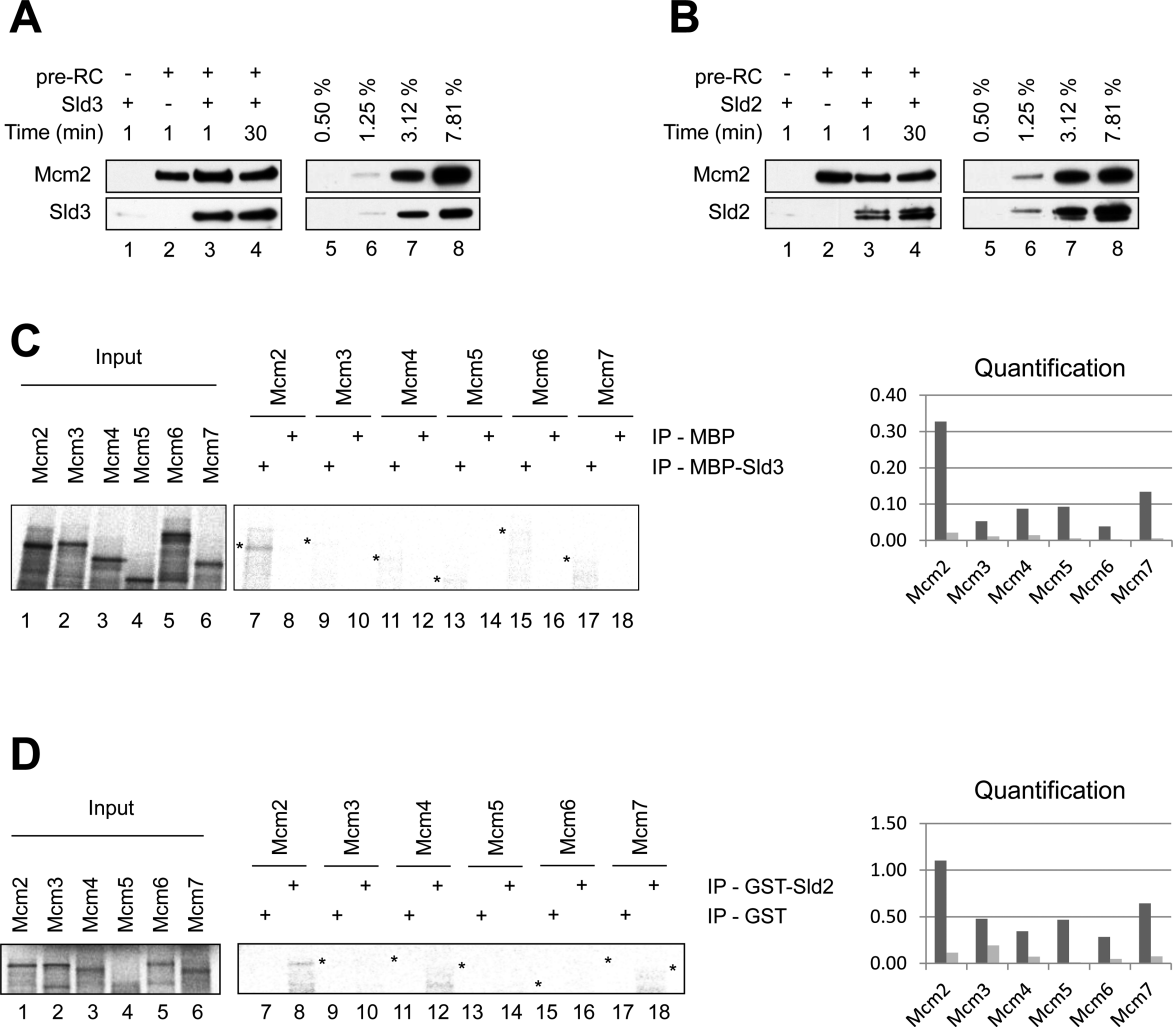
Sld3 and Sld2 interact with the pre-RC via Mcm2. (**A**) Sld3 binds specifically to the pre-RC and the interaction is constant over 30 min of binding time. This interaction was analyzed using the gel-filtration based pre-RC assay. (**B**) Sld2 binds specifically to the pre-RC and the interaction improves over 30 min of binding time. (**C**) Sld3 interaction analysis with Mcm subunits. MBP-Sld3 (400ng) and an equimolar amount of MBP, immobilized on magnetic beads, were incubated with S^35^ labeled *in vitro* transcribed and translated Mcm proteins, washed, separated by SDS-PAGE, analyzed by autoradiography and quantified using Multi Gauge (FUJI) and plotted as % binding. A 5% input was used. (**D**) Sld2 interaction analysis with Mcm subunits. GST-Sld2 (400 ng) and an equimolar amount of GST, immobilized on magnetic beads, were incubated with S^35^ labeled *in vitro* transcribed and translated Mcm proteins, washed, separated by SDS-PAGE, analyzed by autoradiography and quantified using Multi Gauge (FUJI) and plotted as % binding. A 5% input was used. (A and B) Lanes 5–8 show a dilution series representing % of total protein (40 nM), which were added into the reactions.

The MCM2–7 double-hexamer represents a landing platform for a large number of proteins during the G1- to S-phase transition and facilitates pre-IC assembly. Recently, this reaction has been reconstituted with purified proteins (7), but how complex assembly of individual factors at the pre-RC occurs, and equally how complex formation can be inhibited, is only poorly understood. We reasoned that the stepwise addition of purified proteins (Supplementary Figure S1A) could reveal novel mechanisms of complex assembly. Initially, we used the gel-filtration based system to study the binding of purified Sld3 to the pre-RC. We observed that Sld3 was recruited to DNA in a pre-RC dependent manner and that this complex was stable over a time of 30 minutes (Figure 2A). However, when DNA or Cdc6 were removed from the reaction pre-RC formation failed and no Sld3 eluted from the gel-filtration column (Supplementary Figure S2A). This experiment indicates that Sld3 binds specifically to the MCM2–7 double-hexamer. Another factor, which has been suggested to be limiting for helicase activation, is Sld2 (13,28). This protein is of low abundance in the cell and works together with Sld3 to recruit Dpb11 during pre-IC formation. Here we observed binding of purified Sld2 to the pre-RC (Figure 2B). These data suggested that Sld3 and Sld2 make direct contacts with the MCM2–7 double-hexamer. Therefore, we asked if these proteins bind to a specific Mcm subunit using a pull-down assay (Figure 2C, D and Supplementary Figure S1B). We observed that *in vitro* transcribed and translated Mcm2 did interact with MBP-Sld3, but not with the MBP control (Figure 2C). Interestingly, we also observed a similar result for GST-Sld2 (Figure 2D), indicating that both Sld3 and Sld2 interact with the Mcm2 subunit. The binding of Sld2 and Sld3 to the pre-RC was almost stoichiometric, however, in case of the pull-down reactions we detected specific, but reduced binding, probably because the individual Mcm subunits do not fold efficiently or other subunits contribute to binding as well.

### Sld2 and Sld3 bind to each other and compete for interactions with the pre-RC

**Figure 3.**
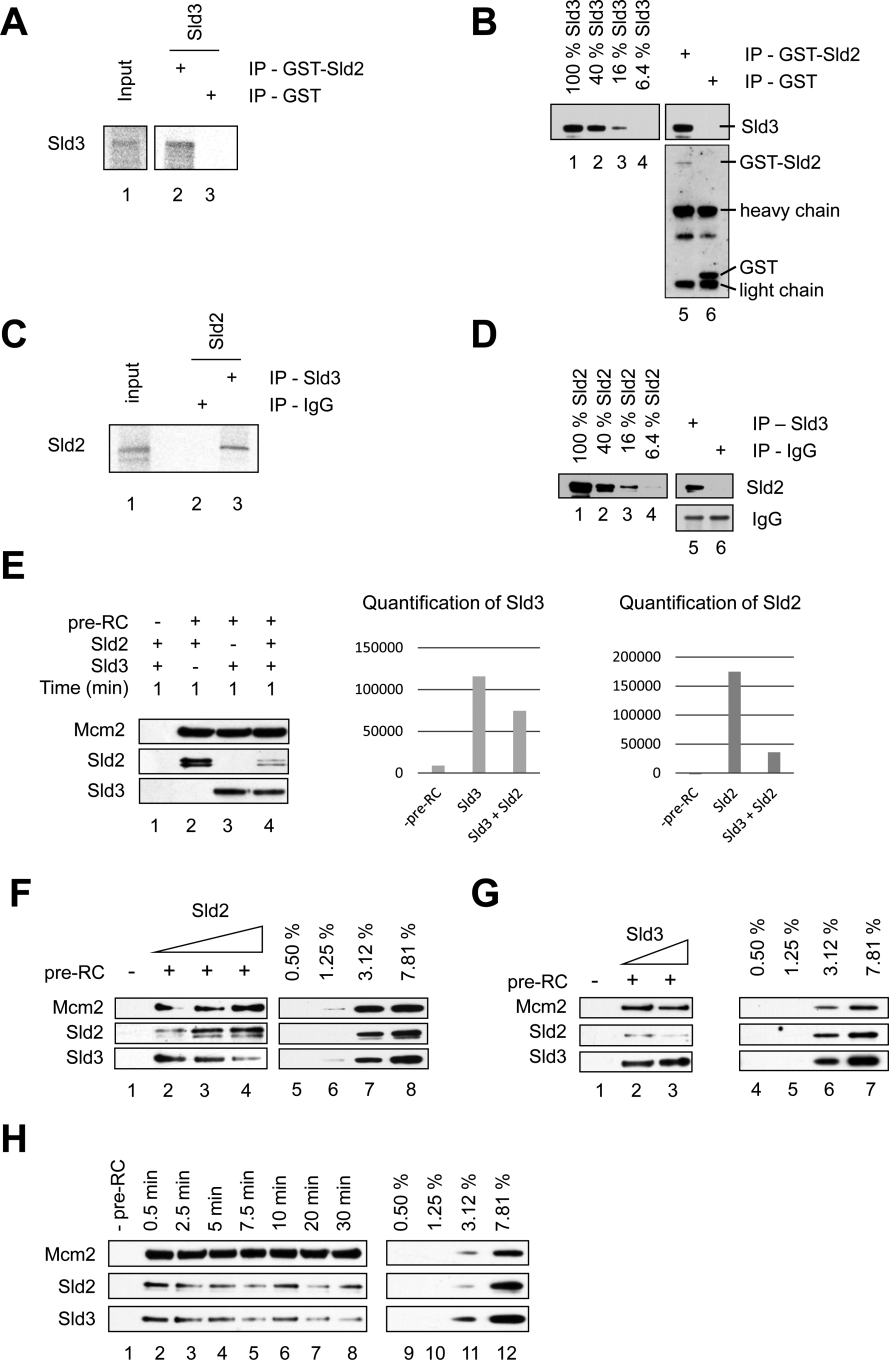
Sld2 and Sld3 compete for pre-RC binding. (**A**) *In vitro* transcribed and translated Sld3 interacts with Sld2. GST-Sld2 (400 ng) and an equimolar amount of GST, immobilized on magnetic beads, were incubated with S^35^ labeled *in vitro* transcribed and translated Sld3, washed, separated by SDS-PAGE and analyzed by autoradiography. A 5% input was used. (**B**) Purified Sld3 interacts with Sld2. GST-Sld2 (400 ng) and GST were immobilized on magnetic beads, incubated with purified Sld3 (500 ng), washed, separated by SDS-PAGE and analyzed by western-blot (lanes 5 and 6). In lanes 1–4 a dilution series is shown representing % of total Sld3 input. (**C**) *In vitro* transcribed and translated Sld2 interacts with Sld3. Sld3 (150 ng) immobilized on anti-Sld3 magnetic beads or control beads, were incubated with S^35^ labeled *in vitro* transcribed and translated Sld2, washed, separated by SDS-PAGE and analyzed by autoradiography. A 5% input was used. (**D**) Purified Sld2 interacts with Sld3. Sld3 (150 ng) immobilized on anti-Sld3 magnetic beads or IgG control beads, were incubated with purified Sld2 (500 ng), washed, separated by SDS-PAGE and analyzed by western-blot. Lanes 1–4; a dilution series representing % of total Sld2 input is shown. (**E**) Sld2 and Sld3 binding to the pre-RC. Sld2, Sld3 or the combination of both was incubated with the pre-RC. The binding of Sld3 and Sld2, corrected for Mcm2 signal, was quantified using Multi Gauge (FUJI) with arbitrary units shown in the quantification. This interaction was analyzed using the gel-filtration based pre-RC assay. (**F**) Sld2 and Sld3 compete with each other for pre-RC binding. Either the same concentration of Sld2 and Sld3 (40 nM each;lane 2) were employed or increasing concentrations of Sld2 (Sld2: 60 nM lane 3 and 90 nM lane 4, with 40 nM Sld3 each). Lanes 5–8 show a dilution series representing % of total protein (40 nM), which were added into the reactions. This experiment has been repeated 3× times with similar results. (**G**) Sld2 and Sld3 compete with each other for pre-RC binding. Either the same concentration of Sld2 and Sld3 40 nM each (lane 2) were employed or 40 nM Sld2/90 nM Sld3 (lane 3), were analyzed for pre-RC binding. Lanes 4–7 show a dilution series representing % of total protein (40 nM), which were added into the reactions. (**H**) Time course analysis of Sld2 and Sld3 association with the pre-RC. After pre-RC formation Sld2 and Sld3 were added and incubated for the indicated time points. The reaction missing the pre-RC proteins was incubated for 1 min. Lanes 9–12 show a dilution series representing % of total protein (40 nM), which were added into the reactions.

Sld2 and Sld3 both interact with Mcm2, indicating that the two proteins could be in close proximity when they are bound to the pre-RC. Thus, one possibility could be that the two proteins interact with each other directly (Figure 3A-D and Supplementary Figure S1B). To address this question we analyzed whether GST-Sld2 could bind to *in vitro* transcribed and translated Sld3. We observed that Sld3 did not bind to the GST negative control, but that it interacted specifically with GST-Sld2, suggesting that Sld2 and Sld3 form a complex (Figure 3A). To confirm this result we repeated the experiment with purified Sld3 and also observed a near stoichiometric interaction between the two proteins (Figure 3B). Consistently, the reverse IP with immobilized Sld3 exhibited specific interactions with *in vitro* transcribed and translated Sld2 (Figure 3C) and a near stoichiometric interactions with purified Sld2 (Figure 3D), demonstrating that Sld2 and Sld3 bind to each other. To address the role of the Sld2-Sld3 interaction within the context of the pre-RC we added Sld2 and Sld3 either individually or in combination to the pre-RC, followed by an one minute incubation (Figure 3E). Here we observed binding of Sld2 and Sld3 individually. However, when the proteins were added together reduced binding of Sld2 and Sld3 was observed, which was more apparent for Sld2 where a ∼4-fold reduction was observed. This could mean that Sld2 and Sld3 compete for Mcm2 binding. To understand if this is indeed the case we added increasing concentrations of Sld2 and a fixed amount of Sld3 to the pre-RC and analyzed their binding (Figure 3F). Indeed, increasing Sld2 in the reaction led to a displacement of Sld3 (Figure 3F, lanes 2–4), and increasing Sld3 led to a displacement of Sld2 (Figure 3G, lanes 2 and 3). To understand if the observed interactions change with prolonged incubation we performed time resolved assays. We observed a decrease of Sld2 and Sld3 binding within the timeframe of 0.5 to 30 minutes (Figure 3H). In summary, these results indicate that Sld2 and Sld3 are competing with each other for pre-RC binding, a mechanism that can limit Sld2 and Sld3 interactions with the MCM2–7 double-hexamer.

### DDK modulates Sld2/Sld3 interaction with the pre-RC

**Figure 4.**
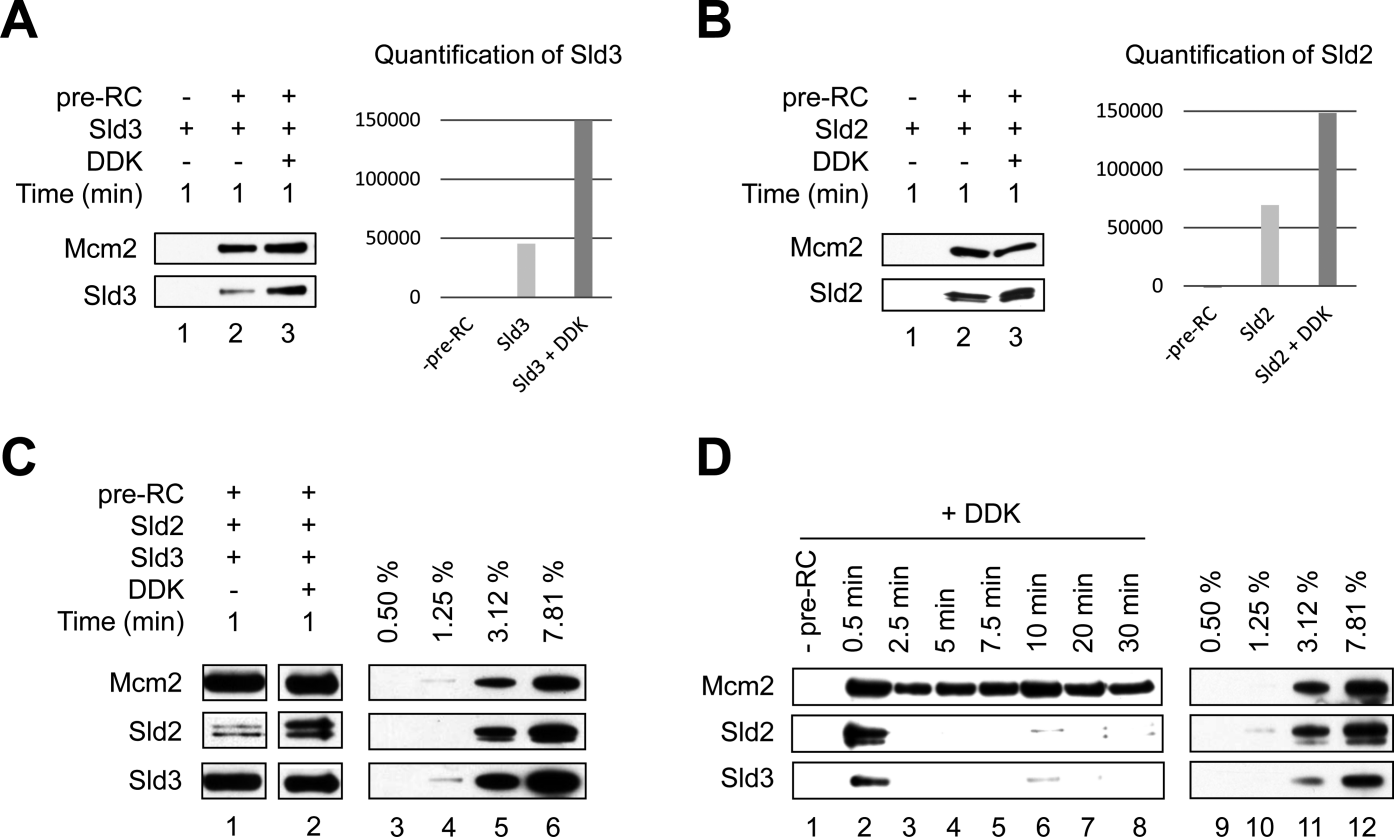
DDK regulates Sld2 and Sld3 interactions with the pre-RC. In all experiments 40 nM of each protein was used unless otherwise stated, besides DDK (5 nM). (**A**) DDK slightly improves Sld3 interactions with the pre-RC. This interaction was analyzed using the gel-filtration based pre-RC assay and the binding of Sld3 corrected for Mcm2 signal was quantified using Multi Gauge (FUJI) with arbitrary units shown in the quantification. (**B**) DDK improves Sld2 interactions with the pre-RC. The binding of Sld2 corrected for Mcm2 signal was quantified using Multi Gauge (FUJI) with arbitrary units shown in the quantification. (**C**) DDK stabilizes Sld2 within the Sld2/Sld3-pre-RC complex. (**D**) Time course analysis of Sld2 and Sld3 association with the pre-RC in the presence of DDK. After pre-RC formation Sld2, Sld3 and DDK were added and incubated for the indicated times. The reaction omitting the pre-RC proteins (-pre-RC) was incubated for 1 minute. (C) Lanes 3–6, (D) lanes 9–12 show a dilution series representing % of total protein (40 nM), which were added into the reactions.

DDK is known to be involved in early steps of helicase activation and has been shown to phosphorylate Mcm2, Mcm4 and Mcm6 (29,30). Furthermore, DDK has been shown to support the recruitment of Sld3 to replication origins in the context of a yeast extract and a reconstituted system (6,7). We wanted to ask if DDK may influence the interaction of Sld2 or Sld3 with the pre-RC. We purified DDK (Supplementary Figure S1A) and verified its activity in the context of the double-hexamer and Sld3 (Supplementary Figure S1C and S1D) (29,31). We observed that DDK-mediated phosphorylation led to a ∼3-fold increased binding of Sld3 to the pre-RC (Figure 4A, compare lane 2 with lane 3), while binding of Sld2 was ∼2-fold improved (Figure 4B, compare lane 2 with lane 3). Interestingly, when Sld2 and Sld3 were added together, DDK also stabilized Sld2 within the Sld2/Sld3-pre-RC complex (Figure 4C). To understand if the Sld2/Sld3-pre-RC complex is stable over time in the presence of DDK, we repeated the experiment and performed a time resolved assay (0.5–30 min). We observed that an initial Sld2/Sld3-pre-RC complex formed in the presence of DDK within 0.5 minutes (Figure 4D, lane 2), but this complex was only short-lived (Figure 4D, lanes 3–8). These results show that DDK enhances initial recruitment of Sld2 and Sld3 to the pre-RC. However, the Sld2/Sld3-pre-RC complex becomes readily destabilized, indicating that DDK induces anti-cooperative interactions that restrict the long term-stability of the Sld2/Sld3-pre-RC complex.

### Sld7 interacts with Sld2 and Sld3

**Figure 5.**
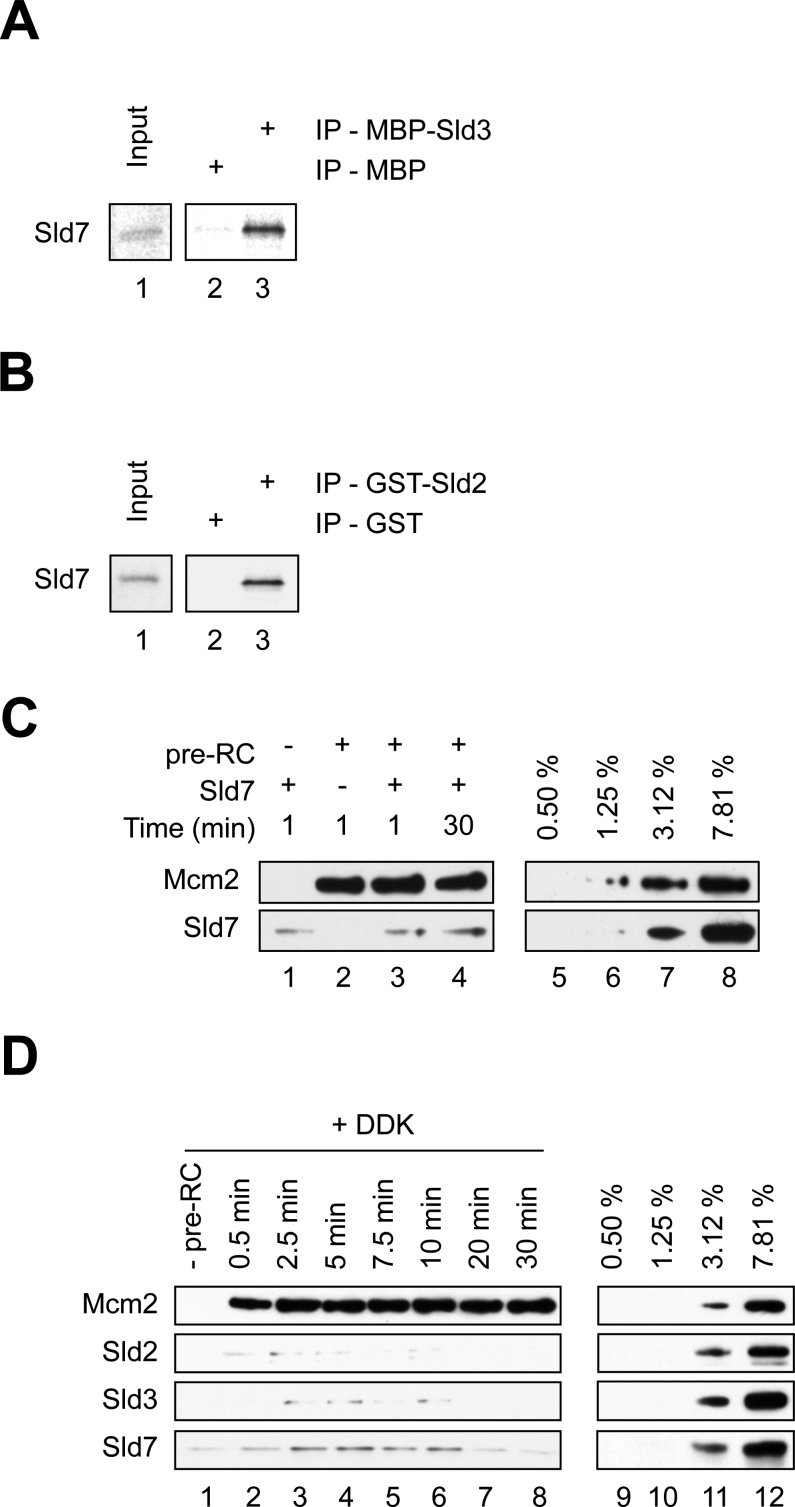
Sld7 mediated interactions with Sld2, Sld3 and the pre-RC. (**A**) *In vitro* transcribed and translated Sld7 interacts with Sld3. MBP-Sld3 (400 ng) and an equimolar amount of MBP, immobilized on magnetic beads, were incubated with S^35^ labeled *in vitro* transcribed and translated Sld7, washed, separated by SDS-PAGE and analyzed by autoradiography. A 5% input was used. (**B**) *In vitro* transcribed and translated Sld7 interacts with Sld2. GST-Sld2 (400 ng) and an equimolar amount of GST, immobilized on magnetic beads, were incubated with S^35^ labeled *in vitro* transcribed and translated Sld7, washed, separated by SDS-PAGE and analyzed by autoradiography. A 5% input was used. (**C**) Sld7 does not bind specifically to the pre-RC. This interaction was analyzed using the gel-filtration based pre-RC assay. (**D**) Time course analysis of Sld2, Sld3 and Sld7 association with the pre-RC in the presence of DDK (5 nM). After pre-RC formation Sld2, Sld3, Sld7 and DDK were added and incubated for the indicated time points. The reaction omitting the pre-RC proteins (-pre-RC) was incubated for 1 min. (C) lanes 5–8, (D) lanes 9–12 show a dilution series representing % of total protein (40 nM), which were added into the reactions.

Sld7 is a small nonessential protein which has been shown to form a complex with Sld3 (15). Since Sld7 interacts with Sld3, we wondered if Sld7 influences Sld2/Sld3/pre-RC complex formation. Initially, we wanted to verify the interaction between Sld7 and Sld3 in the context of purified proteins and also find out whether Sld7 and Sld2 interact (Figure 5A-B and Supplementary Figure S1B). We bound MBP-Sld3 or MBP to anti-MBP beads and incubated these with *in vitro* transcribed and translated Sld7. We observed an interaction between Sld7 and MBP-Sld3, but not with MBP (Figure 5A). In a separate experiment with GST-Sld2 and GST beads we observed that Sld7 interacted specifically with GST-Sld2 (Figure 5B). These data indicate that Sld2 and Sld3 both interact with Sld7. Then we tested if Sld7 binds to the pre-RC in a specific manner. We observed that Sld7 was binding similarly to the pre-RC and the negative control lacking the pre-RC (Figure 5C, compare lane 1 with lanes 3 and 4), suggesting that Sld7 does not bind the pre-RC directly. Next we wanted to address if Sld7 alters Sld3/Sld2-pre-RC complex formation in the presence of DDK. Using time resolved assays we were unable to detect a stable Sld2/Sld3/Sld7-pre-RC complex formation (Figure 5D), while experiments performed in the absence of Sld7 showed an initial Sld2/Sld3-pre-RC complex (Figure 4D, lane 2). These data indicate that Sld7 can also help to limit Sld2 or Sld3 interactions with the pre-RC, potentially by inducing a repressive conformation in Sld2 and/or Sld3.

### Sld3, DDK and Sld2 can promote Cdc45 recruitment to the pre-RC

**Figure 6.**
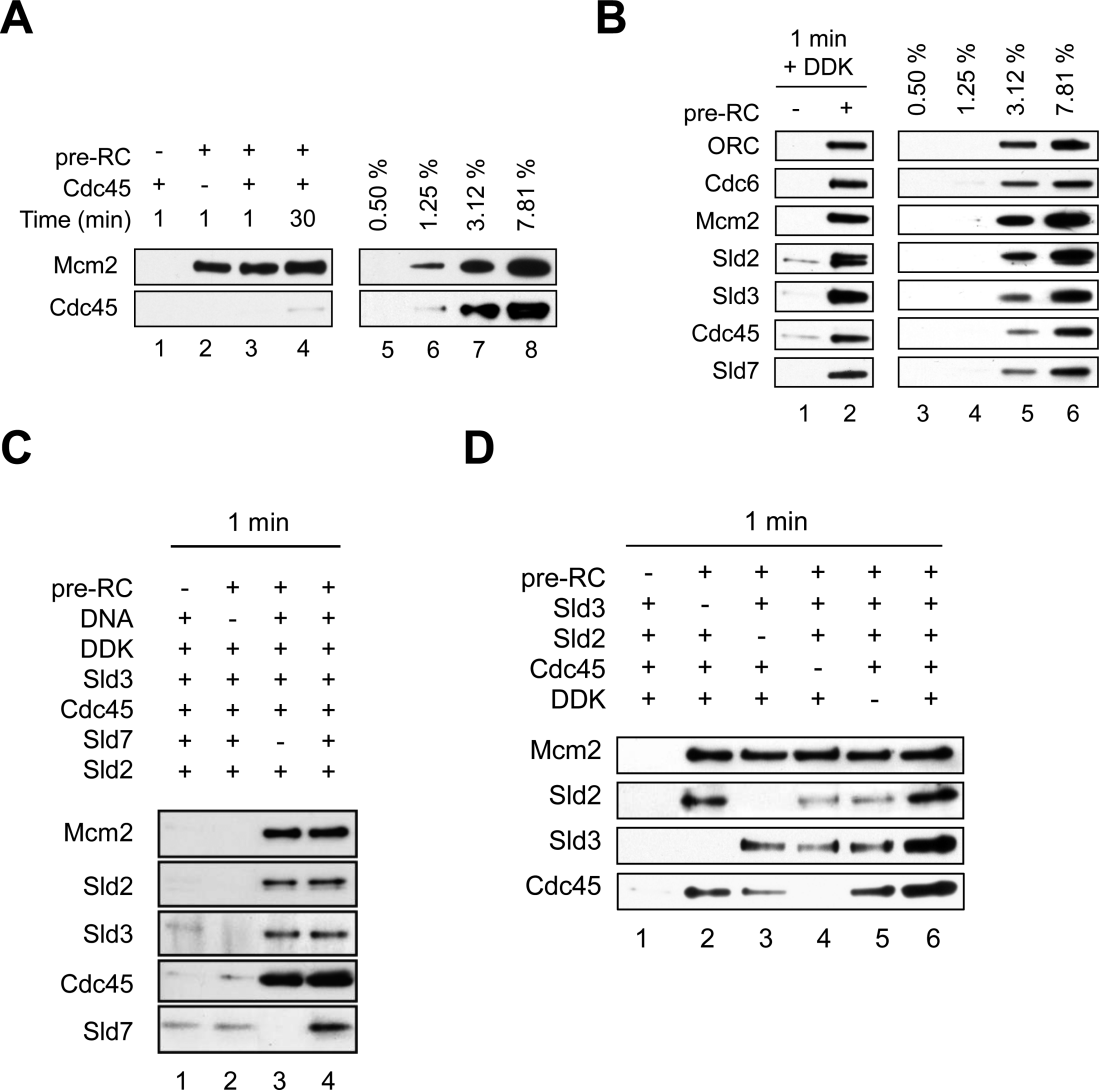
Sld3, Sld2 and DDK induce stable interactions of Cdc45 with the pre-RC. In all experiments 40 nM of each protein was used unless otherwise stated, besides DDK (5 nM). (**A**) Cdc45 interacts weakly with the pre-RC. We observed only after prolonged incubation Cdc45 interactions with the pre-RC. This interaction was analyzed using the gel-filtration based pre-RC assay. (**B**) Efficient Cdc45 interaction with the pre-RC in the presence of Sld3, Sld2, Sld7 and DDK. (**C**) In the absence of DNA no complex formation is observed and Sld7 is not required for Sld2/Sld3/Cdc45-pre-RC complex formation. This experiment was performed in the presence of DDK. (**D**) Sld2, Sld3 and DDK stimulate stable interactions of Cdc45 with the pre-RC. This experiment was performed in the presence of Sld7. (A) Lanes 5–8, (B) lanes 3–6 show a dilution series representing % of total protein (40 nM), which were added into the reaction.

We wondered if Cdc45 could interact with the Sld3/Sld2–pre-RC complex, as Cdc45 is known to interact with Sld3. To address this question we initially asked if Cdc45 on its own could bind to the pre-RC. We observed no interaction after 1 minute of incubation and only sub-stoichiometric binding of Cdc45 to the pre-RC after 30 minutes (Figure 6A, lanes 3 and 4). Next we asked if Sld2, Sld3, Sld7 and DDK could facilitate Cdc45 binding to the pre-RC. When we combined all these factors, we observed efficient Cdc45 recruitment (Figure 6B) to the pre-RC and the resulting Sld2/Sld3/Sld7/Cdc45–pre-RC complex contained near stoichiometric amounts of each component. Importantly, in the absence of DNA we observed no proteins in the elution (Figure 6C, lane 2), highlighting that the observed complex does not represent a non-specific aggregate. Moreover, Sld7 was part of the Sld2/Sld3/Sld7/Cdc45–pre-RC complex. However, removing Sld7 from the reaction had no influence on the recruitment of Cdc45 or other factors (Figure 6C, lane 3), which is not unexpected, as Sld7 is not essential *in vivo*. Importantly, ORC, which stays partially attached with DNA after the gel-filtration (Figure 1C), was by itself not able to promote complex formation (Supplementary Figure S2B). To better understand the requirements of Cdc45 recruitment, we tested complex formation in the absence of individual factors. Indeed, we observed that Sld3 and DDK were sufficient for initial Cdc45 recruitment (Figure 6D, lane3). We suggest that this Cdc45 recruitment could reflect the *in vivo* situation, where weak interaction of Cdc45 with replication origins has been observed in *Saccharomyces cerevisiae* (12). We observed enhanced Cdc45 in the presence of Sld2 (Figure 6D, lane 6). This may reflect a greater stability of the complex in the context of the salt wash employed here. More so, we observed in the presence of Sld7 and Sld2 Cdc45 interaction with the pre-RC (Figure 6D, lane 2). However,*in vivo* Sld7 performs its function in the context of Sld3 and Sld2 performs its function in the context of polymerase ϵ, GINS and Dpb11. To remove all these factors appears impossible and therefore it is difficult to test whether cellular Sld2 could directly contribute to Cdc45 recruitment. Clearly, in the absence of individual factors we observed less efficient Cdc45-pre-RC interactions indicating that the proteins act in a cooperative manner for complex formation (Figure 6D).

### Sld3 and Sld2 stabilize Cdc45 on DNA

**Figure 7.**
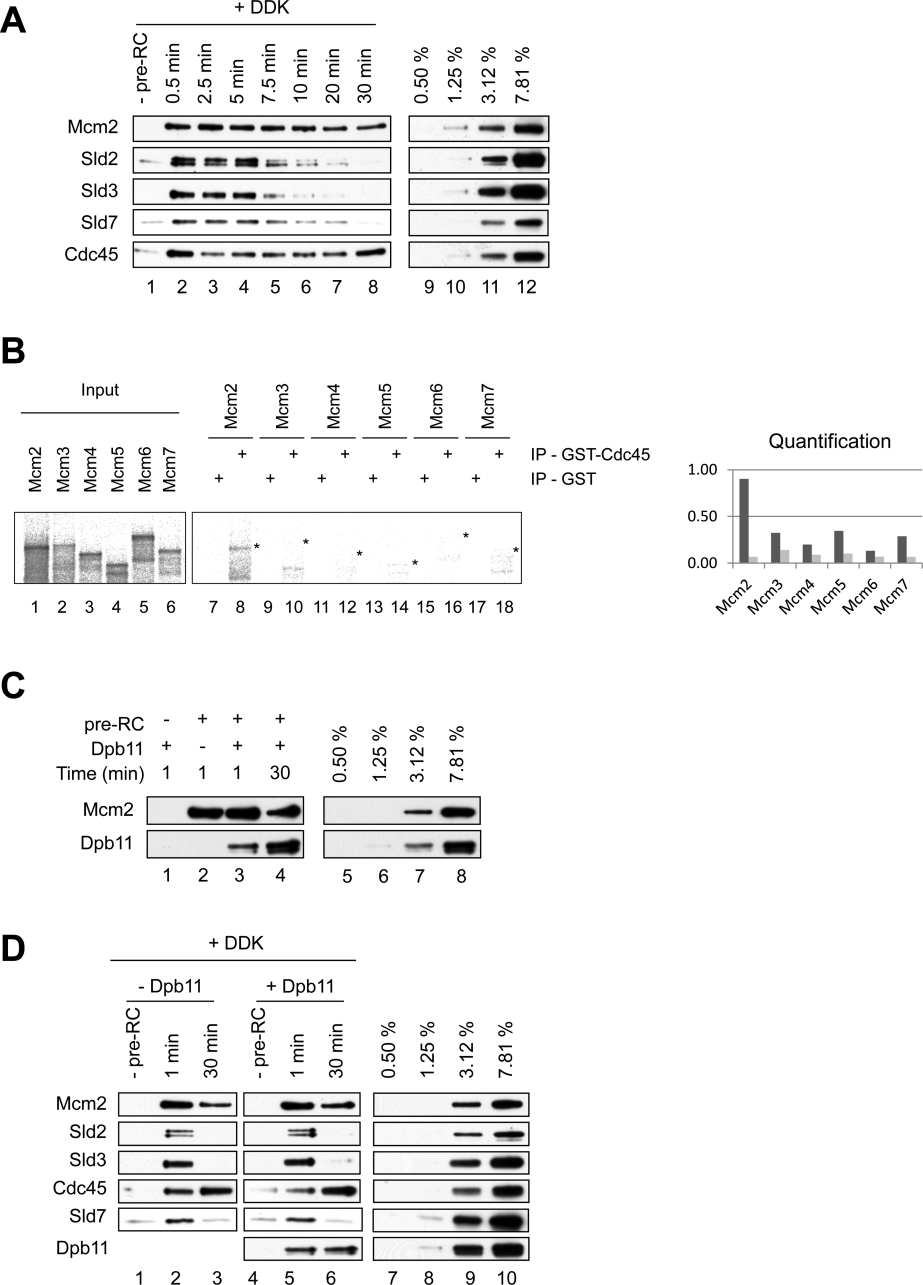
Sld3 and Sld2, but not Dpb11, are important for stable Cdc45 interactions with the pre-RC. (**A**) Time course analysis of Cdc45, Sld3, Sld2 and Sld7 association with the pre-RC in the presence of DDK (5 nM). After pre-RC formation Cdc45, Sld3, Sld2, Sld7 and DDK were added and incubated for the indicated times. The reaction omitting the pre-RC proteins (-pre-RC) was incubated for 1 minute. The 30 min time point shows Sld3, Sld2 independent Cdc45 interactions with the pre-RC. (**B**) Cdc45 interaction analysis with Mcm subunits. GST–Cdc45 (400 ng) and an equimolar amount of GST, immobilized on magnetic beads, were incubated with S^35^ labeled *in vitro* transcribed-translated Mcm proteins, washed, separated by SDS-PAGE, analyzed by autoradiography and quantified using Multi Gauge (FUJI) and plotted as % binding. A 5% input was used. (**C**) Dpb11 binds specifically to the pre-RC and the interaction improves over 30 min of binding time. (**D**) Dpb11 does not influence Cdc45 recruitment or loading at the pre-RC. Reactions were incubated with or without Dpb11 for 1 min, to observe Cdc45/Sld2/Sld3/Sld7-pre-RC complex formation, or for 30 min, to observe Sld2/Sld3/Sld7 independent Cdc45–pre-RC interactions. These reactions contained DDK (5 nM). (A) Lanes 9–12, (C) lanes 5–8, (D) lanes 7–10 show a dilution series representing % of total protein (40 nM), which were added into the reactions.

We observed that Cdc45 recruitment to the pre-RC was promoted by Sld3, DDK and Sld2. However, since the Sld3/Sld2–pre-RC complex was highly unstable in the absence of Cdc45 (Figure 4D), we wondered if Cdc45 could alter complex stability. To address this question we performed time resolved assembly reactions (Figure 7A). We observed rapid and stable Sld3/Sld2/Sld7/Cdc45–pre-RC complex formation. Indeed, the binding of Mcm2 and Cdc45 was relatively constant, while Sld3, Sld2 and Sld7 became destabilized after 5 minutes of incubation. Although this destabilization occurred only after prolonged incubation, it shows that Sld3, Sld2 and Sld7 are not essential for maintenance of the Cdc45-pre-RC complex. As Cdc45 does not bind to the pre-RC on its own (Figure 6A), the data suggests that DDK, Sld3 and Sld2 together promote a structural change in MCM2–7 or Cdc45, which allows formation of a stable Cdc45–pre-RC complex. The observation that Cdc45 recruitment was associated with Sld3 and Sld2 release could suggest that Cdc45 and Sld3/Sld2 compete for MCM2–7 binding. Therefore we asked which Mcm subunit interacts with Cdc45. We observed that Flag–Cdc45 did bind to Mcm2 (Figure 7B and Supplementary Figure S1B), which is consistent with previous observations in *Drosophila* (8). As Sld2 and Sld3 also contact Mcm2, it appears possible that the binding of Cdc45 to Mcm2 promotes the displacement of Sld2 and Sld3.

### Dpb11 binds to the pre-RC, but has no influence on Cdc45 recruitment or loading

Sld3, Sld2, Sld7, Dpb11, Cdc45 and DDK are in low abundance in the cell (13). We observed DDK dependent binding of Sld3, Sld2, Sld7 and Cdc45 to the pre-RC, but it is unclear if Dpb11 is also involved in the process. Currently it is not known if Dpb11 binds the pre-RC on its own or whether Dpb11 binding is strictly dependent on other factors. Therefore we analyzed whether Dpb11 could interact with the pre-RC. Interestingly, we observed that Dpb11 binds readily to the pre-RC and this interaction was maintained over time (Figure 7C). Based on this result Dpb11 could modify the recruitment of Cdc45 or the stability of the Sld3/Sld2/Sld7/Cdc45–pre-RC complex and therefore we performed recruitment assays in the presence of all of these proteins. As shown in Figure 7D, Cdc45 recruitment was not significantly influenced by Dpb11. Furthermore, in contrast to Sld3, Sld2 and Sld7, Dpb11 was not released from the complex after 30 minutes of incubation (Figure 7D, lane 6). Thus, DDK acts specifically on Sld3, Sld2 and Sld7, suggesting that they function as a unit. Our data show that Dpb11 can be recruited independently of Sld3 and Sld2 to the pre-RC. Moreover, Sld3 and Sld2 were not stabilized in the presence of Dpb11. We propose that *in vivo* Dpb11 recruitment to the pre-RC is regulated by interactions with polymerase ϵ, GINS and CDK. However as these factors are absent from our reactions, we could uncover a direct Dpb11–pre-RC interaction. These data are consistent with the idea that Dpb11 functions primarily during CDK dependent complex formation and has no influence on Cdc45 recruitment (6,7,16,17).

### A structural change in MCM2–7 is sufficient to promote Cdc45 recruitment to the pre-RC

**Figure 8.**
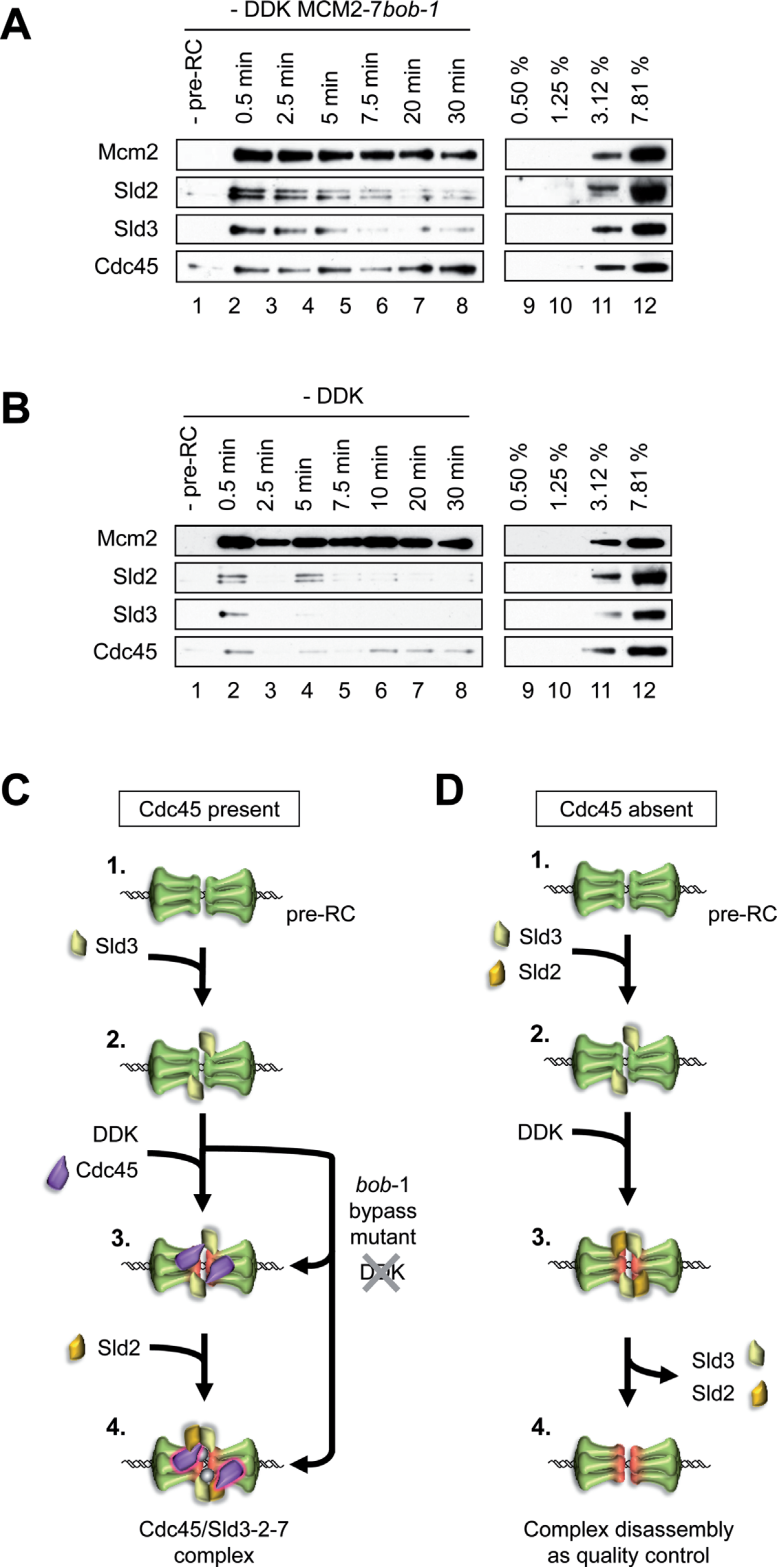
DDK regulates Cdc45-pre-RC complex assembly. (**A**) An MCM2–7 *bob-1* mutant promotes DDK independent Cdc45/Sld3/Sld2/Sld7–pre-RC complex formation. Time course analysis of Cdc45, Sld3, Sld2 and Sld7 association with the pre-RC MCM2–7 containing *bob-1* in the absence of DDK. After MCM2–7 *bob-1* mediated pre-RC formation, Cdc45, Sld3, Sld2 and Sld7 were added and incubated for the indicated time points. The reaction omitting the pre-RC proteins (-pre-RC) was incubated for 1 minute. (**B**) DDK is required to induce stable Cdc45-pre-RC interactions. Time course analysis of Cdc45, Sld2, Sld3 and Sld7 association with the pre-RC MCM2–7 in the absence of DDK. After pre-RC formation Cdc45, Sld3, Sld2 and Sld7 were added and incubated for the indicated time points. The reaction omitting the pre-RC proteins (-pre-RC) was incubated for 1 minute. (**C**) A model of DDK regulated Cdc45 recruitment to the pre-RC. [1] The MCM2–7 double-hexamer is the landing platform for complex formation during S-phase. [2] Sld3 can interact with the pre-RC. [3] DDK and Sld3 are required for Cdc45 recruitment to the pre-RC. [4] Sld2 can stabilize the Cdc45-Sld3-pre-RC complex in the context of the wash conditions employed here. After prolonged incubations Sld3/Sld2/Sld7 become destabilized and reveal a Cdc45-pre-RC complex. The MCM2–7 *bob-1* mutant can bypass the DDK requirement. (**D**) A model explaining how CMG complex formation is inhibited in the absence of Cdc45. [1] The MCM2–7 double-hexamer is the landing platform for complex formation during S-phase. [2] Sld2 and Sld3 compete in the absence of DDK for pre-RC interactions. [3] DDK promotes cooperative Sld2/Sld3-pre-RC binding, but this complex is only short lived [4]. In the absence of Cdc45, Sld2/Sld3 cannot form a stable complex with the pre-RC. Thus, these limiting factors can be redirected to other locations where Cdc45 can induce complex assembly. (A and B) Lanes 9–12 show a dilution series representing % of total protein (40 nM), which were added into the reactions.

We observed that DDK is required for Sld2 and Sld3 dependent Cdc45 recruitment to the pre-RC. One possibility is that a DDK induced structural change in MCM2–7 is sufficient to promote complex formation (32–34). To address this question, we asked if the DDK bypass mutant *bob-1* can promote DDK independent Cdc45 recruitment to the pre-RC (32). Initially, we verified that MCM2–7 *bob-1* functions for pre-RC assembly (Supplementary Figure S3A). Then, a time resolved Cdc45 recruitment assay showed DDK independent Cdc45/Sld3/Sld2-pre-RC complex formation with MCM2–7 *bob-1* (Figure 8A). Importantly, Cdc45 does not bind by itself to the pre-RC, which contains MCM2–7 *bob-1* (Supplementary Figure S3B and C). A time resolved assay with *wild type* MCM2–7 showed reduced Cdc45/Sld3/Sld2–pre-RC formation (Figure 8B) in the absence of DDK. Thus, these experiments show that a structural change in MCM2–7 is crucial for Cdc45 recruitment and stable Cdc45–pre-RC interactions.

## DISCUSSION

Pre-IC formation is a very important step during initiation of DNA replication, as it leads to the assembly and activation of a processive helicase. This unique process does not exist in bacteria or viruses and is in general only poorly understood. Here, we analyzed the first steps in pre-IC formation using purified proteins.

Using this approach we studied complex assembly and mechanisms that restrict pre-IC formation. In the absence of DDK pre-IC formation is completely blocked, while in the absence of CDK Sld3 and Cdc45 interact with the pre-RC (7). Two models could be proposed, either the pre-RC cannot interact with any pre-IC factor or pre-IC proteins engage in interactions that render them refractory to pre-IC formation. We have now observed that Sld3, Dpb11 and Sld2 can specifically interact with the pre-RC, suggesting that refractory interactions limit complex formation. Indeed, we observed competitive interactions between Sld2 and Sld3 for the pre-RC, while the addition of Sld7 and DDK reduced the binding even further. Our data indicate that Sld2, Sld3 and Sld7 can interact with each other (Figures 3 and 5), suggesting that these interactions interfere with their binding to the pre-RC. Then again, it was observed that mutations affecting DDK dependent phosphorylation of Mcm2 interfere with Sld3 release during pre-IC formation causing lethality *in vivo*, indicating that complex disassembly is as important as complex assembly (35). How Dpb11 binding to the pre-RC is prevented is currently unclear, but it is tempting to speculate that integration into the pre-loading complex (pre-LC), containing polymerase ϵ-GINS–Dpb11–Sld2, hinders its binding. So why do these inhibitory interactions exist in the first place? Sld2 and Sld3 are limiting factors in the cell. Currently it is unclear how these factors become distributed to allow the correct temporal and spatial replication initiation program. We suggest that these inhibitory interactions serve as a quality control step to promote a specific program of pre-IC assembly. Indeed, similar inhibitory interactions have been observed during pre-RC formation, here MCM2–7 interactions with ORC/Cdc6 are blocked in the absence of Cdt1. Indeed, these obstacles can be overcome by binding of Cdt1 to MCM2–7 or by removal of an inhibitory domain in Mcm6 (36). Thus inhibitory interactions can limit complex formation, especially when a specific component is missing.

Crucially, any inhibitory force need to be overcome during pre-IC formation. Our data show that a network of cooperative interactions drive complex assembly. It is clear that Cdc45 does interact very poorly with the MCM2–7 double-hexamer, but the addition of Sld3 and DDK leads to recruitment, which reflects the *in vivo* observation where some Cdc45 recruitment occurs already in G1 phase of the cell cycle, prior to S-phase dependent strong Cdc45 recruitment (12). Moreover, this finding is consistent with recent *in vitro* experiments using yeast extracts and purified proteins (6,7), which also showed DDK and Sld3 dependent recruitment of Cdc45. Additionally, Sld3, Sld2, Sld7 and DDK together do not bind to pre-RC, but Cdc45 addition greatly stabilizes the complex. Therefore, Cdc45 must alter the structure of the Sld3/Sld2–pre-RC complex to overcome the inhibitory interactions, indicative of a cooperative Cdc45–Sld3–Sld2–pre-RC binding mode. Similar binding models have been observed in before e.g. Cdt1 binding to MCM2–7 induces structural changes in the MCM complex that are essential for Cdt1/MCM2–7 interactions with ORC/Cdc6 (36).

Surprisingly, during the course of our experiments we observed that the Sld3/Sld2/Cc45-pre-RC complex was more stable than the Sld3/Cdc45-pre-RC complex. One possibility is that Sld2 could directly promote Cdc45 recruitment, as it was found that *Schizosaccharomyces pombe* Sld2, GINS and CDK promote Cdc45 recruitment (37). However, no evidence for such a mechanism has been observed in budding yeast (6,7). Moreover, genetic interactions between Sld2 and Cdc45 have been described, but no physical interactions between Sld2 and Cdc45 have been reported (38). It is possible that the stringent salt wash we performed in our assays may have uncovered a reduced salt stability of the Sld3/Cdc45–pre-RC complex in comparison to the Sld2/Sld3/Cdc45–pre-RC complex. In the context of our data it is conceivable that Sld3 and Sld2 induce structural changes in the pre-RC, which allow more salt resistant Cdc45-MCM2–7 interactions. A detailed structural analysis will be necessary to test this hypothesis in the future. Finally, we suggest that Sld2 functions *in vivo* in the context of polymerase ϵ, GINS and Dpb11 to enhance or stabilize Cdc45-MCM2–7 interactions and CMG formation.

Interestingly, we observed two networks of interactions that participate in pre-IC assembly: the first one connects Mcm2 with Sld3, Cdc45 and Sld2. Indeed, Cdc45 is known to interact with Mcm2 in *Drosophila* (8), but interactions between Mcm2 and Sld3 and Sld2 have previously not been observed. We suggest that Cdc45 interactions with Mcm2 could facilitate the release of Sld2 and Sld3. In addition, it appears possible that these proteins could modify a putative Mcm2/Mcm5 DNA exit gate during pre-IC formation (39). The second network is centered on Sld3. We have now found that Sld2 and Sld3 can interact with each other directly, consistent with genetic interactions observed in *S. pombe* (40). Similarly, Sld7, which is known to bind to Sld3 (15), was found in this study to interact also with Sld2. Thus, these interactions imply that a trimetric Sld7/Sld2/Sld3 complex could exist. We suggest that the Sld2 interactions with Sld7 and Sld3 could function for recruitment of the pre-LC to the Cdc45–Sld3–pre-RC complex.

Finally, our results show that a DDK bypass mutant, Mcm5 *bob-1*, allows Cdc45 recruitment in the absence of DDK. These data suggest that a structural change in MCM2–7 is sufficient for stable Cdc45/Sld2/Sld3/Sld7–pre-RC formation. DDK alters the binding mode of Sld3-Sld2, promoting cooperative interactions specifically in the context of Cdc45, identifying a mechanism for DDK dependent helicase activation. Importantly, once the Cdc45/Sld3/Sld2/Sld7–pre-RC complex is established, Sld3 and Sld2 became over time sensitive to the high salt wash, while the Cdc45–pre-RC interaction is salt resistant. Since Cdc45 does not bind to the pre-RC by itself, these data indicate that Sld3 and Sld2 promote structural changes in the Cdc45–pre-RC complex to facilitate stable Cdc45–pre-RC interactions.

### Models describing DDK dependent reactions for regulated complex formation

The product of pre-RC formation is the MCM2–7 double-hexamer and this complex represents a platform for pre-IC formation (Figure 8C-1). We observed that Sld3 can interact with the pre-RC (Figure 8C-2). In the presence of DDK, Cdc45 can bind to the pre-RC (Figure 8C-3). Cooperative interactions stabilize a Cdc45/Sld3/Sld2/Sld7–pre-RC complex (Figure 8C-4). We observed that Sld2, Sld3 and Sld7 are not required for maintenance of the Cdc45–pre-RC complex, suggesting that the Sld2/Sld3/Sld7 proteins remodel the MCM2–7 complex to promote Cdc45 stabilization. A DDK bypass mutant, Mcm5 *bob-1*, can promote Cdc45 recruitment and loading in the absence of DDK, indicating that a structural change in MCM2–7 is required for Cdc45/Sld2/Sld3/Sld7–pre-RC formation. In the absence of Cdc45 a number of inhibitory mechanisms block complex formation at the pre-RC (Figure 8D-1). Sld3 and Sld2 interact with each other in solution. When Sld2 and Sld3 were added to the pre-RC we observed that these proteins were competing for pre-RC binding, resulting in a somewhat dominant Sld3-preRC complex. These competing interactions restrict the association of Sld2 and Sld3 with the pre-RC (Figure 8D-2). DDK activity induces cooperative Sld3/Sld2-pre-RC interactions (Figure 8D-3). However, if Cdc45 is missing the Sld2/Sld3-pre-RC complex becomes rapidly destabilized (Figure 8D-4). We propose that complex disassembly functions as a quality control mechanism to redistribute Sld2 and Sld3 to sites of preferred complex formation, which could regulate the correct spatial and temporal pattern of DNA replication.

## Supplementary Material

SUPPLEMENTARY DATA
