# Supplementary material for: A reconstituted system reveals how activating and inhibitory interactions control DDK dependent assembly of the eukaryotic replicative helicase
Source: Nucleic Acids Res. 2015 Sep 3;43(21):10238–50. doi: 10.1093/nar/gkv881 (PMC4666391; doi:10.1093/nar/gkv881)
Supplement: SUPPLEMENTARY DATA [file supp_43_21_10238__index.html]

A reconstituted system reveals how activating and inhibitory interactions control DDK dependent assembly of the eukaryotic replicative helicase — SUPPLEMENTARY DATA 

# A reconstituted system reveals how activating and inhibitory interactions control DDK dependent assembly of the eukaryotic replicative helicase

## SUPPLEMENTARY DATA

- SUPPLEMENTARY DATA
