# Supplementary material for: A reconstituted system reveals how activating and inhibitory interactions control DDK dependent assembly of the eukaryotic replicative helicase
Source: Nucleic Acids Res. 2015 Sep 3;43(21):10238–50. doi: 10.1093/nar/gkv881 (PMC4666391; doi:10.1093/nar/gkv881)
Supplement: SUPPLEMENTARY DATA [file supp_gkv881_nar-01335-d-2015-File003.pdf]

Supplementary Data

A RECONSTITUTED SYSTEM REVEALS HOW ACTIVATING AND INHIBITORY INTERACTIONS CONTROL DDK DEPENDENT ASSEMBLY OF THE EUKARYOTIC REPLICATIVE HELICASE

M. Carmen Herrera, Silvia Tognetti, Alberto Riera, Juergen Zech, Pippa Clarke, Alejandra Fernández-Cid and Christian Speck

Figure S1 (related to main Figure 2). Proteins used in this study

Figure S2 (related to main Figure 2 and 6). Complex formation is specific

Figure S3 (related to main Figure 8). MCM2-7 bob-1 analysis

Supplementary Material & Methods

Table S1. Oligonucleotides

Table S2. Plasmids

Table S3. Antibodies

Supplementary References

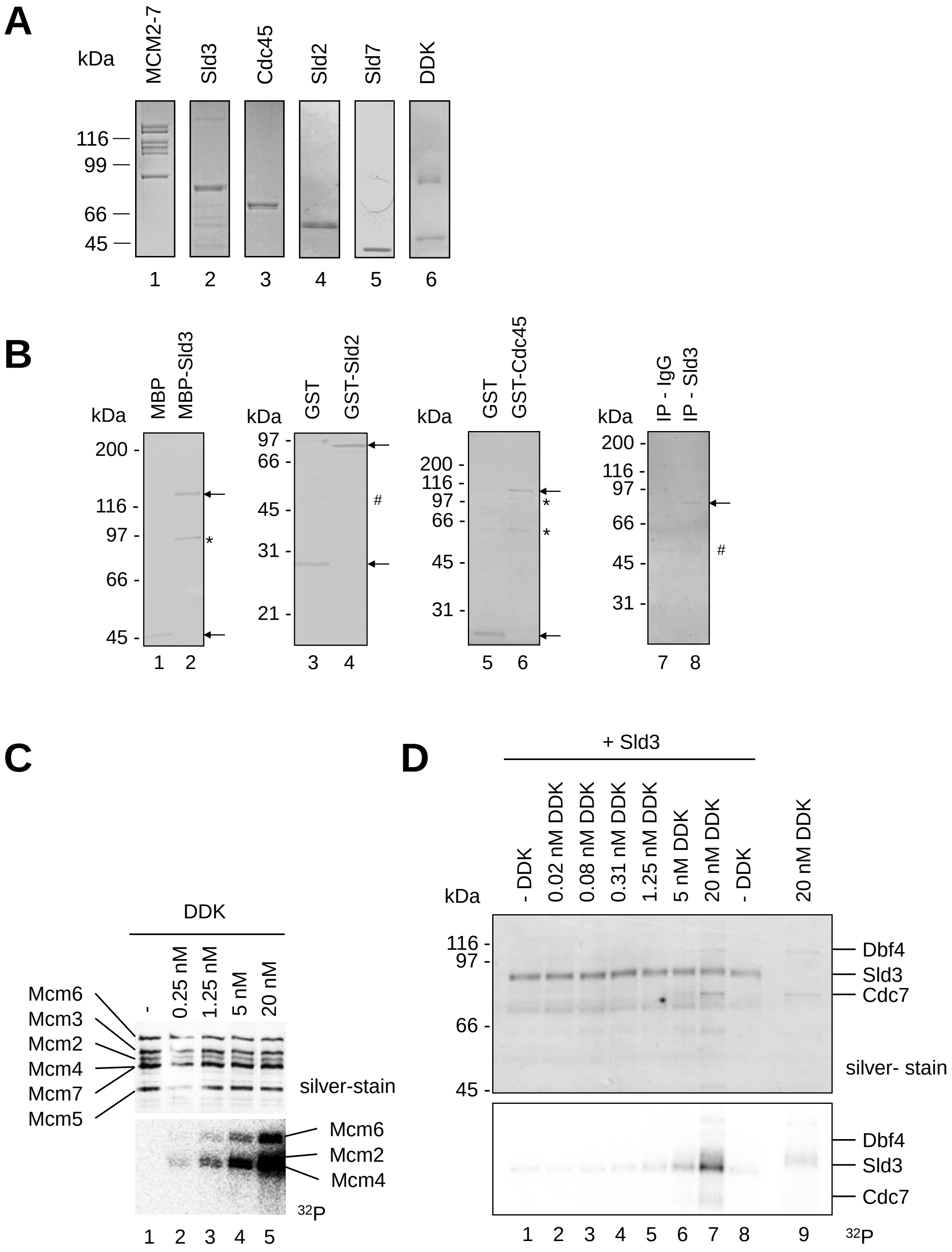

Figure S1 (related to main Figure 2). Proteins used in this study. (**A**) Purified proteins. Please note that DDK is a dimer consisting of Cdc7 and Dbf4. (**B**) Immobilized proteins used for pull down reactions. An arrow marks the specific proteins, the asterisk degradation products and the hash indicates the heavy chain of the antibody. (**C**) Analysis of DDK activity in the context of the loaded MCM2-7 double-hexamer. (**D**) Analysis of DDK activity in the context of Sld3.

A

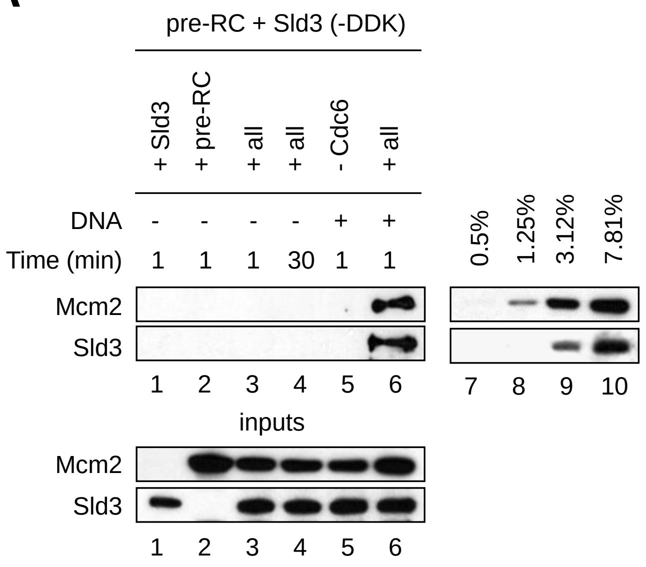

B

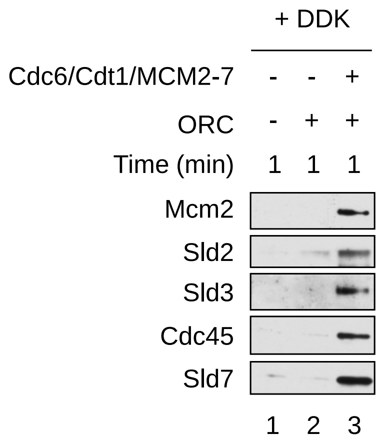

Figure S2 (related to main Figure 2 and 6). Complex formation is specific. (**A**) Cdc6 and DNA are essential to promote Sld3 recruitment to the pre-RC. "+ all" indicates the presence of both, Sld3 and the pre-RC proteins. The load indicates the presence of the factors before loading onto the gel-filtration column. (**B**) ORC does not support Cdc45 recruitment. 40 nM of each protein was used.

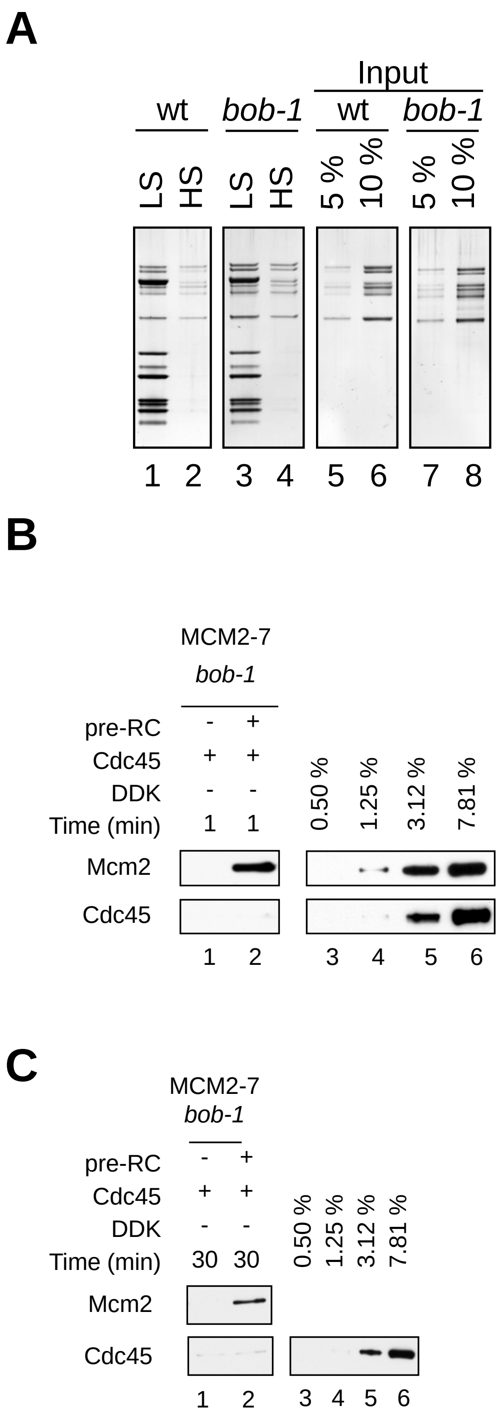

Figure S3 (related to main Figure 8). MCM2-7 *bob-1* analysis. (**A**) MCM2-7 *bob-1* promotes efficient MCM2-7 association in the presence of low salt (LS) and high salt (HS) resistant MCM2-7 loading. This assay was performed with the magnetic bead based method (2). (**B**) Cdc45 interacts weakly with the pre-RC MCM2-7 *bob-1*. We observed only very weak Cdc45 interactions with pre-RC. This interaction was analyzed using the gel-filtration based pre-RC assay. (**C**) Prolonged interactions of Cdc45 with the pre-RC MCM2-7 *bob-1* indicate weak nonspecific Cdc45 interactions with DNA. (B and C) Lanes 3-6 show a dilution series representing % of total protein (40 nM), which were added into the reactions.

Supplementary Material & Methods

Oligonucleotides, plasmids and antibodies

Oligonucleotides, plasmids and antibodies used in this study are listed in Table S1, S2 and S3, respectively.

Cloning

The coding sequence of Sld2 and Dpb11 were amplified by PCR with primers (CS1144, CS1145 and CS1260, CS1261, respectively) that added NotI and EcoRI restriction sites. The PCR products were cloned into pGEX 6P1 vector.

The coding sequence of Sld7 was amplified by PCR with primers CS1653 and CS1654, adding NotI and BamHI restriction sites and cloned into pGEX-6P1 (GE Healthcare).

The coding sequence of Sld3 was amplified by PCR with primers CS785 and CS818, adding BamHI and SalI restriction sites and cloned into pESC-HIS (Agilent Technologies). This construct was then modified by insertion of an N-terminal MBP tag, amplified from pMALC2x with primes CS921 and CS922. The primers also coded for a PreScission protease site and a myc tag and BamHI restriction sites.

For yeast expression, the coding sequence of Cdc45 (missing the stop codon) was amplified by PCR with primers CS561 and CS572, adding NotI and SpeI restriction sites and cloned in pESC-HIS, pESC-LEU, pESC-TRP and pESC-URA vectors (Agilent Technologies). The stop codon was deleted in order to have a C-terminal FLAG tag.

The coding sequence of Cdc45 was amplified by PCR with primers CS591 and CS592 adding BamHI and NotI restriction sites and cloned in pGEX 6P1 vector (GE Healthcare) for bacterial expression.

MBP-Prescission-Dbf4 and Cdc7 were cloned into pESC-TRP (Stratagene). The *dbf4* gene was amplified from genomic DNA of the *S. cerevisiae* strain S288C with the primers CS1052 and CS1053 via SmaI/NheI. The MBP-PreScission gene was amplified from pCS245 with primers CS921 and CS922 and cloned in before the dbf4 gene using a BamHI site. The cdc7 gene was amplified from genomic DNA of the *S. cerevisiae* strain S288C with the primers CS1058 and CS1059 and cloned via NotI/SpeI the vector. The resulting plasmid is pCS313.

Bacterial protein expression

The respective plasmids were transformed in BL21 Codon Plus RIL *E. coli* competent cells (Agilent). The cells were grown in Terrific Broth supplemented with appropriate antibiotics at 37°C to an OD600 of 1. Protein expression was then induced by addition of 0.5 mM IPTG for 5h at 16°C.

Yeast protein expression

Yeast strain AS499 (*MATa. bar1Δ, leu2-3,-112, ura3-52, his3-Δ200, trp1-Δ-63, ade2-1 lys2-801, pep4*) was transformed. The yeasts were grown overnight in selective medium at 30°C. The proteins were expressed as described previously (3).

Protein purification

Sld3 purification

Cells were lysed in a freezer mill. The cells were resuspended in buffer AS [50 mM HEPES/NaOH pH 8.1, 0.5 M NaCl 10% glycerol, 0.1% NP40, 1mM DTT] plus complete protease inhibitor cocktail (PIC) (Roche), extracted on a rotating wheel for 2h at 4 °C and centrifuged at 27,600 g for 1 h at 4 °C. The extract was incubated for 2h at 4 °C with Amylose Resin (NEB). The beads were afterwards rinsed with 10 column volumes buffer AS + PIC and washed twice with 20 cv Buffer AS + PIC for 30 min and once with 20 cv Buffer AS without PIC for 30 min. MBP-myc-Sld3 was eluted at that stage with buffer AS + 10 mM Maltose. To elute myc-Sld3, instead, beads were incubated with 1 cv buffer AS (without protease inhibitors and PreScission protease for 2 h at 4 °C and rinsed afterwards additionally with 1 cv buffer AS. Elutions were diluted to 0.1 M NaCl, bound 30 min to SP-Sepharose (GE Healthcare). The resin was rinsed once with 10 cv buffer BS [50 mM HEPES/NaOH pH8.1, 0.1 M NaCl 10% glycerol, 0.1% NP40, 1mM DTT] and once with 10 cv buffer CS [50 mM HEPES/NaOH pH 8.1, 0.2 M NaCl 10% glycerol, 0.1% NP40, 1mM DTT]. The protein was eluted in buffer AS.

Sld2 purification

The cells were lysed by sonication in buffer A [50 mM PIPES (pH 6.5), 500 mM AS, 10 mM $MgCl_2$, 5 mM DTT, and 1% Triton, 10% Glycerol, 2 mM ATP]. The fusion protein was bound to glutathione agarose (Sigma) in buffer A at 4 °C for 2 h, and then the protein was eluted by addition of PreScission Protease (GE Healthcare) for 2h at 4 °C. The eluate was diluted with 1 volume buffer A with no salt and bound to SP Sepharose (GE Healthcare). Sld2 was eluted with buffer B [50 mM PIPES (pH 6.5), 500 mM AS, 5 mM DTT, and 0.1% Triton, 10% Glycerol].

Cdc45 purification

The protein was purified as previously described (4). To remove the FLAG peptide the sample was incubated for 30 min with DEAE beads (GE Healthcare), the resin was extensively washed and the protein was eluted with purification buffer containing 500 mM KCl.

Sld7 purification

The cells were lysed by sonication in buffer C [50 mM HEPES-KOH (pH 7.5), 300 mM NaCl, 10 mM $MgCl_2$, 1 mM DTT, and 1% Triton, 10% Glycerol, 2 mM ATP]. Ammonium sulphate was added to a final concentration of 250 mM to the extract. Nucleic acids were precipitated from the cell extract by the addition of 30 µl 10 % polymin P/HCl (pH 6.5) per ml of extract. GST-Sld7 was precipitated by adding 0.4 g fine powdered ammonium sulphate per ml of extract. The fusion protein was bound to glutathione agarose (Sigma) in buffer C at 4 °C for 2 h, and then Sld7 was eluted by addition of PreScission Protease (GE Healthcare) for 2h at 4 °C. The eluate was concentrated using a centricon concentrator (Millipore).

DDK purification

Cells were lysed in a freezer mill. The resulting extract was incubated with amylose resin (NEB) pre-equilibrated with buffer DK [50 mM Hepes-KOH pH 7.5, 400 mM NaCl, 1 mM EDTA, 1 mM EGTA, 10% glycerol, 0.02% NP40, 1 mM DTT] and supplemented with proteases and phosphatases inhibitors for 2 h at 4°C. The protein was eluted by cleaving the MBP tag via addition of PreScission protease for 2 h at 4°C. The eluate was incubated with Glutathione agarose for 2 h to remove the PreScission protease. The GST flow-through was collected and concentrated by centricon (Millipore).

Dpb11 purification

The cells were lysed by sonication in buffer D [50 mM PIPES (pH 6.5), 100 mM NaCl, 3 mM DTT, and 1% Triton, 10% Glycerol]. Ammonium sulphate was added to a final concentration of 250 mM to the extract. Nucleic acids were precipitated from the cell extract by the addition of 45 µl 10 % polymin P/HCl (pH 6.5) per ml cell extract. Protein from the supernatant was precipitated by adding 0.35 g fine powdered ammonium sulphate per ml of extract. The fusion protein was bound to glutathione agarose (Sigma) in buffer D at 4 °C for 2 h, and then Dpb11 was eluted by addition of PreScission Protease (GE Healthcare). The eluate was diluted with 1 volume buffer D with no salt and bound to SP Sepharose (GE Healthcare). Dpb11 was eluted with buffer E [50 mM PIPES (pH 6.5), 500 mM NaCl, 5 mM DTT, 10 mM $MgCl_2$, 0.1% Triton, 10% Glycerol].

Competitor DNA

Two complementary oligonucleotides (CS1126 and CS 1127, table S1) were incubated in buffer M [100 mM Tris-HCl (pH 7.9), 100 mM NaCl, 1 mM DTT, 10 mM $MgCl_2$] for 5 min at 95 °C and then slowly cooled down to RT over 30 min. The hybridized oligos were concentrated using Microcon (Millipore) to a concentration of 5 µM.

In vitro transcription and translation

To generate ^{35}S-Met-labelled proteins, the coding sequences of the genes were amplified using a sense primer and anti-sense primers, as suggested by the supplier's instruction (Promega). To compensate for different methionine content additional methionines were incorporated into the anti-sense primer. 400 ng of the resulting PCR product was used as template in the TNT-T7 Quick for PCR DNA system (Promega), combined with 20 µl of TNT Quick Master Mix and 20 µCi of ^{35}S-Met (Perkin-Elmer) in a total volume of 25 µl and incubated for 90 min at 30°C with mixing.

Sld3 pull-down reactions

<u>Immobilisation (MBP-Sld3):</u> MBP-Sld3 (400 ng) and an equimolar amount of MBP were immobilized on anti-MBP antibody (NEB) beads (protein G - Sigma) for 15 min at 24°C with mixing in 50 µl of Sld3 buffer CS [pH 7.5] followed by three washes with 100 µl of buffer I (225 mM K-Glu, 1% BSA)].

<u>Immobilisation (Sld3):</u> Sld3 (150 ng) was immobilized on anti-Sld3 318 beads (protein G) for 15 min at 24°C with mixing in 50 µl of Sld3 binding buffer followed by three washes and used for pull downs, together with IgG control beads.

Pull-down: *In vitro* translated proteins or purified proteins (0.5 μg) were incubated with MBP or MBP-Sld3 beads for 2h (*in vitro* translated proteins) or 1 h (purified proteins) with mixing at 4°C in 500 μl (*in vitro* translated proteins) or 200 μl (purified proteins) of buffer I (225 mM K-Glu, 1% BSA); afterwards the beads were washed three times with 200 μl of buffer I (225 mM K-Glu) and separated by SDS/PAGE followed by phosphor-imager analysis (*in vitro* translated proteins) or Western blot (purified proteins).

Sld2 pull-down reactions

Extract preparation: The pellets of 50 ml of GST and GST-Sld2 cultures were resuspended in 2.5 ml of Sld2 buffer A. 0.1 mg/ml of lysozyme and 25 U/ml Benzonase (Merck) were added and the mixture was incubate on ice for 30 min. The extract was sonicated, the supernatant separated by centrifugation and aliquoted.

Immobilisation (GST-Sld2): GST-Sld2 (400 ng) and an equimolar amount of GST were immobilized by incubating 50 μl of GST and GST-Sld2 containing extracts with anti-GST antibody (Sigma) beads (protein G – GE Healthcare) for 1h at 4°C with mixing in 100 μl of Sld2 buffer A followed by three washes with 100 μl of buffer I (225 mM K-Glu, 1% BSA).

Pull-down: *In vitro* translated proteins or 0.5 μg of purified proteins were incubated for 2 h (*in vitro* translated proteins) or 1 h (purified proteins) with mixing at 4°C in 500 μl (*in vitro* translated proteins) or 200 μl (purified proteins) of buffer I (225 mM K-Glu, 1% BSA); afterwards the beads were washed three times with 200 μl of buffer I (225 mM K-Glu) and analysed by SDS/PAGE followed by phosphorimaging analysis (*in vitro* translated proteins) or Western blot (purified proteins).

Cdc45 pull-down reactions

Extract preparation: The pellets of 50 ml of GST and GST-Cdc45 culture were resuspended in 2.5 ml of Cdc45 binding buffer (C45), then 0.1 mg/ml of lysozyme were added and the mixture was incubated on ice for 30 min. The extract was sonicated and the supernatant separated by centrifugation and aliquoted.

Immobilisation and pull-down: GST-Cdc45 (400 ng) and an equimolar amount of GST were immobilized by incubating 50 μl of GST and GST-Cdc45 containing extracts with anti-GST antibody (Sigma) beads for 1h at 4°C with mixing in 100 μl of buffer C45 and used for pull downs as described for Sld2.

Table S1. Oligonucleotides

Primers name	Usage	Gene	Primers sequence
CS1144	Cloning	Sld2	ATGACGCGGCCGCTCACCTTCTTCCCCATCGT
CS1145	Cloning	Sld2	AGTCTGGAATTCATGTACTCATTTGAACTGGACAAATTG
CS1260	Cloning	Dpb11	CCGCTCGAGTCAAGAATCTAATTCCTTTGTCTGATTTC
CS1261	Cloning	Dpb11	ATAAGAATGCGGCCGCATGAAGCCCTTTCAAGGAATAAC
CS1653	Cloning	Sld7	TATTCAGTGGATCCATGTCACGGAAATTATGCACAC
CS1654	Cloning	Sld7	CATTATATATGCGGCCGCTCATGATTTGGTAAAGAGC
CS785	Cloning	Sld3	CGCGGATCCATGGAAACATGGGAAGTCATAGC
CS818	Cloning	Sld3	ATGGCCGACGTCGACCTATGTGGATTCTGGAGCAAATAA
CS561	Cloning	Cdc45	GCTAGCGGCCGCATGTATTATGGAATCAGCCAGTTTAG
CS572	Cloning	Cdc45	GCTAACTAGTCCTAACAATCCACTCAAGGTCAGC
CS591	Cloning	Cdc45	GACTGCGGATCCATGTATTATGGAATCAGCCAGTTTAGC
CS592	Cloning	Cdc45	GACTGCGCGGCCGCTTATAACAATCCACTCAAGGTCAGC
CS1052	Cloning	Dbf4	TCCTCCCCCGGGATGGTTTCTCCAACGAAAATGA
CS1053	Cloning	Dbf4	CTACTACTAGCTAGCCTATATTTGAAATCTGAGATTTTC
CS1058	Cloning	Cdc7	AAGGAAAAAAGCGGCCGCATGACAAGCAAAACGAAGAATA
CS1059	Cloning	Cdc7	CTAGCTAGACTAGTCTATTCAGATATTAGGAGAACAT

CS1126	Competitor DNA	-	TCCTGCTGGAATATACAGATGAACCCGGGACGG GTCGTTCT
CS1127	Competitor DNA	-	AGAACGACCCGTCCCGGGTTCATCTGTATATTC CAGCAGGA
CS1670	*In vitro* translation	Mcm2	*ACTGATC TAATACGACTCACTATAGGG CAT CCACCATG* TCTGATAATAGAAGACGTAGAC
CS1671	*In vitro* translation	Mcm2	AAAAAAAAAAAAAAAAAAAAAAAAAAAAAA TTA GTG ACC CAA GGT ATA AAT TGC
CS1667	*In vitro* translation	Mcm3	ACTGATC TAATACGACTCACTATAGGG CAT CCACCATG GAAGGCTCAACGGGATTTG
CS1674	*In vitro* translation	Mcm3	AAAAAAAAAAAAAAAAAAAAAAAAAAAAAA TCA GAC TCT CCA AAC TTT ATC G
CS1677	*In vitro* translation	Mcm4	ACTGATC TAATACGACTCACTATAGGG CAT CCACCATG TCTCAACAGTCTAGCTCTCCAA
CS1678	*In vitro* translation	Mcm4	AAAAAAAAAAAAAAAAAAAAAAAAAAAAAA TCA GAC ACG GTT ATT CAG GCG
CS1681	*In vitro* translation	Mcm5	ACTGATC TAATACGACTCACTATAGGG CAT CCACCATG TCATTTGATAGACCGGAAATATAC
CS1682	*In vitro* translation	Mcm5	AAAAAAAAAAAAAAAAAAAAAAAAAAAAAA TCA TAC ACC ACT TCT GTA AAT ATT C
CS1685	*In vitro* translation	Mcm6	ACTGATC TAATACGACTCACTATAGGG CAT CCACCATG TCATCCCCTTTTCCAGC
CS1686	*In vitro* translation	Mcm6	AAAAAAAAAAAAAAAAAAAAAAAAAAAAAA TTA GCT GGA ATC CTG TGG TTC
CS1689	*In vitro* translation	Mcm7	ACTGATC TAATACGACTCACTATAGGG CAT CCACCATG AGTGCGGCACTTCCATC
CS1690	*In vitro* translation	Mcm7	AAAAAAAAAAAAAAAAAAAAAAAAAAAAAA TCA AGC GTC TTG TAG ATC GAT ATC AG
CS1766	*In vitro* translation	Sld3	*ACTGATC TAATACGACTCACTATAGGG CAT CCACCATG* GAA ACA TGG GAA GTC ATA G
CS1767	*In vitro* translation	Sld3	AAAAAAAAAAAAAAAAAAAAAAAAAAAAAA CTA TGT GGA TTC TGG AGC AAA TA
CS2044	*In vitro* translation	Sld2	*ACTGATC TAATACGACTCACTATAGGG CAT CCACCATG TAC TCA TTT GAA CTG GAC AAA TTG*

CS2050	*In vitro* translation	Sld2	AAAAAAAAAAAAAAAAAAAAAAAAAAAAA CTA TCA CCT TCT TCC CCA TCG TC
CS2040	*In vitro* translation	Sld7	*ACTGATC TAATACGACTCACTATAGGG CAT CCACCATG* TCA CGG AAA TTA TGC ACA CTA AAT TTT
CS2041	*In vitro* translation	Sld7	AAAAAAAAAAAAAAAAAAAAAAAAAAAAA CTA CAT TCA TGA TTT GGT AAA GAG CTT CAG
CS1764	*In vitro* translation	Cdc45	ACTGATC TAATACGACTCACTATAGGG CAT CCACCATG TAT TAT GGA ATC AGC CAG TTT AG
CS1765	*In vitro* translation	Cdc45	AAAAAAAAAAAAAAAAAAAAAAAAAAAAA TTA TAA CAA TCC ACT CAA GGT C
CS2042	*In vitro* translation	Dpb11	*ACTGATC TAATACGACTCACTATAGGG CAT CCACCATG AAG CCC TTT CAA GGA ATA AC*
CS2043	*In vitro* translation	Dpb11	AAAAAAAAAAAAAAAAAAAAAAAAAAAAA CTA TCA AGA ATC TAA TTC CTT TGT CTG
CS921	MBP-Protease-BamHIb	MBP	CGCGCGGGATCCGGGCCCCTGGAACAGAACTTCCAGGGTCGTATCGATCCCGAGGT
CS922	MBP-Protease-myc-BamHIb	MBP	CGCGCGGGATCCGAGGTCTTCTTCGGAAATCAACTTCTGTTCGGGCCCCTGGAACAGAACTTCCAGGGTCGTATCGATCCCGAGGT

Table S2. Plasmids

**Plasmid name**	**Gene/DNA Sequence**	**Vector**	**Source**
pSC372	ARS1	pUC19	Evrin C *et al*, PNAS. 2009
pCS245	*sld3*	pESC-HIS	This study
pCS8	*cdc45*	pESC-HIS	This study
pCS241	*cdc45*	pESC-LEU	This study
pCS242	*cdc45*	pESC-TRP	This study
pCS243	*cdc45*	pESC-URA	This study
pCS229	*cdc45*	pGEX-6P1	This study
pSC290	*sld2*	pGEX-6P1	This study
pSC486	*sld7*	pGEX-6P1	This study
pSC300	*dpb11*	pGEX-6P1	This study
pCS313	*cdc7/dbf4*	pESC-Trp	This study

Table S3. Antibodies

Antibody name	Antigen	Peptide Sequence		Source
Cdc6	Cdc6	-	mouse	Santa Cruz (SC53218)
ORC1	ORC1	-	mouse	Stillman (SB13)
MCM2	MCM2	-	mouse	Zou and Stillman, Mol Cell Biol. 2000
Sld3 (318)	Sld3	CTKKGLVRRRSKKKTSE	rabbit	This study
Sld3 (418)	Sld3	CKLKGPSMRPKRALKKVND	rabbit	This study
Sld2	Sld2	CRKRPKRRKVIRRLRDNDPETE	rabbit	This study
Sld7	Sld7	THRDELKRISMGSGEEVS	rabbit	This study
Dpb11	Dpb11	CGSIQDKKRTASLEKPMRRQTRNQTKE	rabbit	This study
FLAG	FLAG epitope	-	mouse	Sigma (A8592)
MBP	MBP	-	mouse	NEB (E8032L)
GST	GST	-	mouse	Pierce (P08263)

Supplementary References

1. Remus, D., Beuron, F., Tolun, G., Griffith, J.D., Morris, E.P. and Diffley, J.F. (2009) Concerted loading of Mcm2-7 double hexamers around DNA during DNA replication origin licensing. *Cell*, **139**, 719-730.
2. Evrin, C., Clarke, P., Zech, J., Lurz, R., Sun, J., Uhle, S., Li, H., Stillman, B. and Speck, C. (2009) A double-hexameric MCM2-7 complex is loaded onto origin DNA during licensing of eukaryotic DNA replication. *Proc Natl Acad Sci U S A*, **106**, 20240-20245.
3. Burgers, P.M. (1999) Overexpression of multisubunit replication factors in yeast. *Methods*, **18**, 349-355.
4. Heller, R.C., Kang, S., Lam, W.M., Chen, S., Chan, C.S. and Bell, S.P. (2011) Eukaryotic origin-dependent DNA replication in vitro reveals sequential action of DDK and S-CDK kinases. *Cell*, **146**, 80-91.
